# Wood Chips Versus Barrels in Beer Maturation: Chemical, Sensory, Microbiological, and Operational–Economic Perspectives

**DOI:** 10.3390/molecules31203741

**Published:** 2026-10-18

**Authors:** Maciej Jamiński, Paweł Satora

**Affiliations:** 1 Department of Business Informatics and Management Engineering, Faculty of Management, AGH University of Krakow, Mickiewicza Av. 30, 30-059 Kraków, Poland; 2 Department of Fermentation Technology and Microbiology, Faculty of Food Technology, University of Agriculture, Balicka Street 122, 30-149 Kraków, Poland

**Keywords:** wood chips, beer maturation, wood in brewing, barrel-aged beer, phenolic compounds, sensory profile, wood toasting

## Abstract

Wood contact is a common method for modifying the chemical and sensory properties of beer, although its effects depend strongly on wood form and processing conditions. This narrative review summarises the use of wood chips during beer maturation and compares them with barrel and untreated storage in terms of chemical, sensory, microbiological and operational outcomes. Direct beer studies comparing wood chips, barrels and untreated controls under identical conditions are limited. Consequently, evidence concerning microbiological behaviour, operational performance and some maturation mechanisms is partly derived from barrel studies, model systems, or other alcoholic beverages and interpreted as indirect evidence. The review evaluates the influence of wood species, toasting level, different physicochemical conditions, reuse and beer composition on the extraction of phenolic, furanic, and volatile compounds and their sensory effects. Compared with chips, barrels provide a more complex environment through gradual extraction, micro-oxygenation and, in some beer styles, microbial succession. The review also assesses implementation, sanitation, reuse, costs, consumer acceptance and environmental aspects. Future research should focus on standardized methodologies, direct chip–barrel–control comparisons, microbiological validation, techno-economic analyses, life-cycle assessment and consumer studies.

## 1. Introduction

Maturation in contact with wood is one of the technological approaches used to modify the chemical composition and sensory properties of beer. Wood is not merely a construction material for maturation vessels, but also a source of compounds that can migrate into beer and affect its aroma, taste, color, mouthfeel and physicochemical stability. The principal groups of wood-derived substances relevant to beer include lactones, phenolic acids, phenolic aldehydes, furanic compounds, and volatile phenols. Depending on their concentration and interactions with compounds originating from malt, hops and fermentation, these substances may contribute vanilla, coconut-like, toasted, smoky, spicy and woody sensory notes [1,2]. Consequently, contact with wood should be considered a controlled technological operation capable of modifying the quality and differentiation of the final product.

Traditionally, beer maturation in contact with wood has been associated with wooden barrels and larger wooden vessels. Barrel maturation is a complex process in which the extraction of wood-derived compounds occurs simultaneously with limited oxygen transfer, gradual chemical transformations and, in certain beer categories, microbial activity within the porous structure of the wood. These mechanisms may contribute to the development of complex sensory profiles, particularly in strong, sour, mixed-fermentation, and specialty beers. However, the barrel environment is inherently variable. The final effect may depend on wood species, barrel size, degree of toasting, previous use, residual compounds from the former contents, storage conditions and the microorganisms retained in the internal structure of the vessel. Barrel maturation is also associated with long processing times, substantial space requirements, and difficulties in sanitation and process standardization [3,4].

These limitations have encouraged the development of alternative methods of beer–wood contact, including wood chips, cubes, staves, spirals and wood-derived extracts. Among these forms, wood chips are of particular technological interest because their relatively high surface-area-to-volume ratio promotes rapid contact between wood and beer. Their use also enables more direct control of material-related and process-related variables, including wood species, degree of toasting, dose and contact time [5,6,7]. Wood chips can be introduced into vessels already used in brewery production, which may facilitate small-scale trials and reduce the need for dedicated barrel storage infrastructure.

Nevertheless, wood chips and barrels should not be treated as technologically equivalent systems. Wood chips act predominantly through the extraction of compounds from the wood surface. They may reproduce selected aromatic and flavor characteristics associated with wood maturation, but they do not inherently provide the same oxygen transfer conditions, long-term transformations, or stable microbial niches as wooden barrels. The technological question is therefore not whether wood chips completely replace barrels, but which effects of barrel maturation they can reproduce and under what conditions these effects can be achieved in a repeatable and controlled manner.

Previous studies confirm that the use of wood chips can modify the volatile composition, monophenol concentration and physicochemical properties of beer [5,6,7,8]. However, the magnitude and direction of these changes differ substantially among experiments. This variability results from differences in wood species, geographical origin, particle size, degree of toasting, material dose, contact time, temperature, alcohol concentration, beer style and the stage of production at which wood is introduced. The composition of the base beer is also important because the same wood-derived compounds may be perceived differently in pale, dark, low-alcohol, high-alcohol, sour or strongly hopped beers. Consequently, results obtained for one combination of wood material and beer matrix cannot be directly transferred to another process without considering these interacting variables.

The lack of standardized experimental conditions is one of the principal limitations of current research. Studies use different wood forms, doses, maturation periods, analytical methods and sensory procedures, which restricts direct comparison between results and prevents the formulation of universal technological recommendations. In particular, the literature does not provide a single optimal dose, contact time, or degree of toasting applicable to all beer styles. The technological effect of wood chips should therefore be evaluated as the outcome of interactions between the properties of the wood, the conditions of maturation, and the composition of the beer rather than as the result of a single independent parameter.

Microbiological stability represents an additional issue in the use of wood materials. The porous structure of wood may retain yeasts and bacteria that can contribute either to desirable maturation processes or to product instability. This distinction is particularly important when comparing barrel maturation, in which microbial activity may be an intentional component of product development, with the use of wood chips as a controlled technological additive. In the latter case, the origin, storage, preparation and microbiological quality of the material must be considered as elements of process control. Detailed microbiological mechanisms and their effects on beer quality are discussed in the subsequent sections of this review.

Despite the growing interest in wood-assisted beer maturation, current knowledge remains fragmented. Most studies focus on oak, while information on alternative wood species is less extensive. Direct comparisons of different wood species, degrees of toasting, and doses under identical brewing conditions are uncommon. Evidence concerning operational efficiency, process repeatability, economic implications, and consumer acceptance is also limited. Furthermore, many publications examine wine, spirits or model hydroalcoholic solutions rather than beer. Although such studies provide useful information on extraction mechanisms and wood chemistry, their findings require cautious interpretation because beer differs in pH, ethanol content, raw material composition, and colloidal structure.

Previous reviews have addressed the chemistry of wood-derived compounds, alternative wood species, accelerated ageing, and the broader chemical, sensory and technological mechanisms of wood maturation in alcoholic beverages [1,2,9,10]. However, direct comparisons between wood chips and barrels as distinct technological beer maturation systems are less common, particularly when considering the effects on chemicals, sensory properties, microbiological behaviour, process implementation, sanitation, resource use, economics, consumer response, and environmental implications together. This review therefore takes a comparative approach, treating wood chips and barrels as distinct maturation systems rather than interchangeable sources of wood-derived flavor. Particular emphasis is placed on distinguishing the effects primarily attributable to the extraction of compounds from fragmented wood from those associated with the broader physical, chemical, and biological environment of barrel maturation.

The aim of this review is to systematize current knowledge on the use of wood in beer maturation and to assess the factors determining their chemical, sensory, microbiological, and technological effects. Particular attention is given to wood species, degree of toasting, dose, contact time, temperature, alcohol content, and the properties of the base beer. The review also compares wood chips with barrel maturation and beer produced without direct wood contact in order to determine which effects are specific to rapid extraction from wood fragments and which arise from the more complex barrel environment.

Accordingly, the review addresses four principal questions: how do the properties and forms of wood influence the migration of wood-derived compounds into beer; which process parameters determine the intensity and direction of these changes; to what extent can wood chips reproduce selected effects of barrel maturation; and what technological, microbiological and operational limitations affect their application in brewing practice?

This article is a narrative literature review based on a thematic synthesis of studies concerning beer matured in contact with wood. Literature searches were conducted using Scopus, with the search updated to 1 July 2026. An iterative search strategy was applied using combinations of terms related to the beverage matrix (e.g., “*beer*”, “*ale*”, “*lager*”, “*brewing*”), wood treatment (e.g., “*wood chips*”, “*oak chips*”, “*wood adjunct*”, “*wood aging*”, “*barrel aging*”, “*oak barrel*”, “stave*”, “cube*”), and the investigated outcomes (e.g., “*flavor*”, “*aroma*”, “*sensory*”, “*chemical composition*”, “phenolic*”, “*volatile*”, “*physicochemical*”, “*quality*”, “*microbiology*”, “*process*”, “*cost*”, “*economic*”, “*environmental*”, “sustainab*”, *and* “*life cycle*”). More focused searches were subsequently performed to identify studies directly comparing fragmented wood with barrel maturation and untreated beer, as well as evidence concerning process implementation, sanitation, reuse, economic implications, and environmental performance.

Keyword-based searches were supplemented by natural-language queries addressing the effects of wood chips on beer composition and sensory quality, comparisons between chip and barrel maturation, and the operational, economic and environmental aspects of the two approaches. Full-text searches were additionally used to retrieve methodological and experimental details concerning maturation protocols, extraction, oxidation, sanitation, reuse, and process outcomes. These complementary searches were used for evidence discovery rather than as independent criteria for study inclusion.

Priority was given to studies directly examining wood chips, cubes, barrels or other wood forms in beer production and reporting chemical, physicochemical, sensory or microbiological outcomes. Evidence was interpreted according to its proximity to the research question: (i) direct beer studies using wood chips or other fragmented wood, (ii) direct barrel-aged beer studies, (iii) beer-related experimental or model systems, and (iv) supplementary evidence from wine, spirits, or hydroalcoholic model systems. Evidence from the latter categories was used primarily to explain mechanisms or operational aspects for which direct beer data were unavailable and was not treated as equivalent to direct beer evidence.

The review does not aim to establish universal processing parameters or to provide a quantitative meta-analysis. Substantial heterogeneity in wood species and form, dose, effective contact area, beer composition, maturation temperature and duration, analytical methods and sensory protocols prevents direct quantitative synthesis across the available studies. Instead, the review identifies variables requiring process control, evaluates the comparability and limitations of the available evidence, and highlights areas requiring further experimental, operational, and economic investigation.

## 2. Factors Influencing the Maturation of Beer in Wood

The effect of using wood chips during the maturation of beer depends on a number of material and process factors. The most important of these are the wood species used, the degree of toasting, the dosage of wood used, the contact time with the beer, the temperature, the alcohol content, and the composition of the base beer. These factors influence the rate and extent to which phenolic compounds (like aldehydes and volatile phenols), lactones, and furan compounds are extracted, consequently affecting the intensity of the aroma and flavor profile [1,2,5,6,7].

### 2.1. Wood Species

Oak is the most commonly used and best-described raw material. This is due to its chemical composition, technological availability and long-standing role in the maturation of alcoholic beverages. Oak wood is a source of the compounds responsible for aromas such as vanilla, coconut, spice, toast and wood. Oak lactones, vanillin, eugenol and certain phenolic aldehydes are particularly significant [1,2]. In beer, these compounds can enrich the aromatic profile; however, their impact depends on the style of beer, its alcohol content, extract, bitterness, and the presence of compounds derived from malt, hops, and fermentation [5,6,7].

Only a few studies have directly compared American (*Quercus alba*) and French oak (*Q. petraea* and *Q. robur*) in beer, and outcomes depend on the dose of chips used, toasting, ageing time, beer style and the initial chemistry of the beer [11,12]. Short-contact chip treatments can leave beers with volatile profiles that are very similar to the control group, meaning that differences in oak origin are easiest to detect after longer or more extractive maturation conditions [5,13]. Evidence from direct beer analysis suggests that American oak extracts more phenolic acids than French oak, while French oak can increase the levels of other volatile compounds. In pale and dark beers aged with chips, American oak increased the levels of 3,4-dihydroxyphenylacetic acid, vanillic acid, and syringic acid by approximately three-fold, two-fold, and ten-fold, respectively, compared to French oak. However, malt type still accounted for more overall variation in phenolic compounds than wood origin [14]. These differences were observed under specific experimental conditions and should not be interpreted as universal characteristics of geographically defined American and French oak. Where available, botanical species, toast level, wood dose, contact time and beer matrix should therefore be considered when interpreting comparisons between oak origins. A separate beer flavor study also found that effects of wood origin were real at the sensory and monophenol level and not just an artefact of toasting [6,7].

The maturation of beer in non-oak wood is a topic of emerging scientific interest, but there is currently limited information directly linking the properties of beer aged in different types of non-oak wood (Table 1). The evidence for the use of acacia, cherry, and some Brazilian woods as substitutes for oak in beer aging comes primarily from studies of wood properties [5,9,15,16,17,18].

Acacia (*Robinia pseudoacacia*) is one of the better-characterized non-oak woods investigated directly in beer. In a 38-week barrel ageing experiment, its effects were compared with those of European oak (*Quercus petraea* and *Q. robur*) [11]. Specifically, eugenol and oak lactones were found to be significantly higher in oak-aged beer in comparison to the acacia-aged variant following 38 weeks of aging, with the latter exhibiting only marginal increases. In turn, acacia-aged beer was found to contain higher contents of total polyphenols and β-glucan, leading to different sensory perceptions in comparison to its oak counterpart [11]. Finally, variation in the wood source was also found to impact the bacterial composition of barrel-aged beers, with oak-aged variants possessing significantly higher levels of *Acetobacter* and *Paenibacillus* compared to acacia-aged beer, potentially due to differences in porosity, oxygenation rates, and antimicrobial compounds extracted from the wood [11].

Cherry chips were tested in beer, but proven to only marginally alter volatile composition when macerated for a short time frame at low concentrations, with the control often exhibiting higher overall volatile contents than wood treated variants [5]. Meanwhile, wood materials native to Brazil have been found to successfully produce lager beer aged with Brazil nut (*Bertholletia excelsa*), amburana (*Amburana cearensis*), balsam (*Myroxylon balsamum*), and Cariniana (*Cariniana* sp.) cubes, with trained sensory panels noting increases in complexity over untreated controls [16].

### 2.2. Forms of Wood

Several technological forms of wood can be used in beer maturation, differing in terms of the surface area in contact with the beer, extraction dynamics, process control and the extent to which they influence the sensory profile of the product. The most common methods are using barrels, wood chips, staves, spirals, and aromatic extracts or essences. The form of wood used influences the rate at which phenolic and volatile compounds migrate into the beer, as well as the possibility of micro-oxidation and microbiological transformations [5,6,7].

Barrel maturation is the most traditional form of beer–wood contact. The barrel serves as a source of compounds extracted from the wood, provides an environment of limited oxygen exchange, and acts as a potential microbiological niche. In this setting, the maturation process involves extraction, as well as slow chemical and microbiological transformations, which can enhance the complexity of the aroma and flavor profile. Important factors include the properties of the wood, the barrel’s history, its degree of roasting, maturation time, and the composition of the microbiota present in the wood structure [11,12]. The limitations of this method include long processing times, high storage requirements, the cost of purchasing and maintaining barrels, and difficulty achieving full batch-to-batch consistency.

Wood chips offer a more flexible technological solution. The key feature of wood chips is their large contact surface area relative to their volume, which promotes the extraction of substances responsible for vanilla, coconut, toast, smoke, spice and woody notes. Research into beer production indicates that using wood chips influences the monophenol content, volatile composition, and overall sensory profile of the final product [5,6,7,8]. Compared to barrels, wood chips allow for easier regulation of dosage, contact time, wood species and degree of toasting, increasing control over the intensity of the woody profile. However, precise parameter selection is required, as excessive dosage or contact time can lead to dominance of woody notes, increased astringency and sensory imbalance [6,7].

Wooden staves and spirals are an intermediate solution between barrels and wood chips. They provide a larger surface area for contact than barrel walls, but a smaller one than fine wood chips. This allows for more moderate and stable extraction. They can be used in steel tanks, eliminating the need for barrels while maintaining natural contact between beer and wood. Such solutions are widely studied in wine and spirits’ technology, where their ability to shorten maturation times and reduce process costs has been demonstrated [21,22,23]. In brewing, they can serve a similar function; however, the scope of research into their direct impact on beer is smaller than in the case of wood chips and barrels.

Aromatic extracts and essences allow beer to quickly acquire specific wood-related aromas. While their use reduces processing time and facilitates standardization of aroma intensity, they do not replicate the maturation mechanism associated with natural wood. Unlike wood chips, staves or barrels, essences do not facilitate the gradual extraction of multiple compound groups or the processes of micro-oxidation and microbiological transformation. Therefore, while they can be useful for correcting the aroma profile, their technological and sensory impact is usually more limited than that of natural wood [1,2].

Wood contact systems differ primarily in their dominant maturation mechanisms, degree of process control, and ability to reproduce barrel-specific conditions (see Table 2). Barrels combine extraction with limited oxygen exchange and the potential involvement of microbiota, whereas wood chips primarily intensify the extraction of woody substances. Staves and spirals are intermediate solutions, whereas aromatic essences are primarily used to adjust the aroma profile. Therefore, the choice of wood type should be based on the desired technological effect, the required process time, and the level of control the brewery wishes to maintain over the product profile.

### 2.3. Wood Toasting, Dose of Wood, and Time of Aging

The second key factor is the degree of wood toasting. This thermal process causes the degradation and transformation of wood’s main structural components, primarily lignin and hemicellulose. These transformations result in the formation of compounds that are important for the taste and aroma, such as vanillin, syringaldehyde, coniferyl aldehyde, furfural, 5-methylfurfural, eugenol, guaiacol and other volatile phenols [1,2,24,25,26,27,28,29,30]. This treatment mainly affects the wood surface, meaning that fragments such as chips and cubes have a larger toasted area and usually accelerate extraction compared to whole barrels [14]. Light toasting promotes a more subtle wood profile, whereas medium and heavy toasting increase the contribution of toasted, roasted, smoky and spicy notes. However, excessively intensive thermal treatment or an excessively high wood dose may lead to over-extraction of compounds responsible for bitterness, astringency, and the dominance of woody aromas [26,27,28,29,30]. In lager aged three months with cubes of French oak, levels of aldehydes, esters, higher alcohols, and wood-derived compounds were found to rise over time. Heavily toasted cubes had the highest volume of low-molecular-weight phenolics in the experiment, compared to those aged in barrels and less toasted cubes. Contact with wood also led to higher concentrations of gallic acid, 5-HMF, and furfural than in the bottle-stored variant. In lager matured for three months at 0 °C with French oak cubes, the concentrations of wood-related compounds changed over time. Heavily toasted cubes produced the highest overall concentrations of low-molecular-weight phenolic compounds, but individual compounds did not follow a simple toast-intensity response. For example, furfural decreased during the second month in all treatments and remained at approximately 0.3 mg/L during the third month [20].

The dose of wood chips is also important. Increasing the wood dose generally increases the amount of extractable material available to the beer, although the resulting extraction also depends on particle geometry and effective contact area. This effect may be technologically beneficial when the aim is to rapidly impart characteristics associated with maturation in contact with wood. At the same time, an excessively high dose increases the risk of obtaining an unbalanced profile in which woody, toasted, or smoky aromas dominate over the character of the base beer. For this reason, the dose of wood chips should be selected together with contact time, degree of toasting, and beer style [6,7]. Available beer studies do not support a universal sensory or technological threshold expressed solely as wood dose in g/L. Correia et al. [5] observed only limited and beer-dependent changes after short maceration with 1 g/L of oak or cherry chips, whereas Machado et al. [14] reported compositional changes using 2 g/L under a different beer matrix and maturation protocol. These results should therefore be interpreted within their individual experimental conditions rather than as evidence of a general 1–2 g/L dose–response relationship. Wood dose should be considered together with particle geometry, effective contact area, wood species, toasting, beer composition, and contact time.

Contact time is another parameter determining the chemical composition of the product. In the initial phase of contact with wood, extraction may proceed intensively, especially in the case of wood chips with a large specific surface area. As the process is prolonged, the migration rate of some compounds may decrease, while the risk of accumulation of substances affecting astringency and persistent aftertaste increases [5,6,7]. In barrel-aged sour beer, later time points show lower sugars and higher wood-related com-pounds, organic acids, and microbial phenols, indicating that extraction and biotransformation accumulate together [31]. In full-scale barrel aging, total polyphenols increased over time in some beers; ethanol rose slightly; and glucose, fructose, and sucrose generally declined over the maturation period [32]. Furthermore, extended contact of beer with wood enhances particular aromatic compounds. For example, stout aged in pre-soaked American oak chips for six weeks had a significantly higher content of wood-derived phenolics and volatiles than the control [19]. In a 300-day barrel ageing study of lager beer, ageing progressively modified the sensory and volatile profile, with increasing woody, sweet, and caramel attributes. (Z)-Oak lactone was strongly correlated with ageing time and with woody and sweet odor notes [33].

### 2.4. Temperature and Alcohol Content

Extraction is influenced by temperature, but available studies should be interpreted separately according to the experimental system. Coelho et al. [34] investigated reused wood under elevated-temperature conditions of 30–50 °C; these conditions represent an accelerated experimental treatment rather than conventional beer maturation. Model beer solutions investigated by Sterckx et al. [7] provide mechanistic information on temperature-dependent monophenol extraction but cannot be quantitatively equated with finished beer. In direct beer experiments, Machado et al. [14] used post-fermentation contact at 22 °C, whereas Wyler et al. [20] matured lager with oak material at 0 °C. Collectively, these studies indicate that temperature modifies extraction kinetics, but they do not constitute a single temperature-response series and do not demonstrate that higher maturation temperature necessarily improves sensory quality.

Water and ethanol penetrate the wood structure and facilitate the solubilization and extraction of accessible wood-derived compounds. Chemical transformations of lignin- and hemicellulose-derived constituents may also occur during maturation, but these processes should be distinguished from physical extraction and depend on the treatment and storage conditions [1]. Model work in beer-like solutions showed that monophenol concentrations increased almost linearly with the amount of chips added, and that they were higher at 8–10% ethanol and particularly at low pH and warmer storage temperatures [7]. The strongest mechanistic study specific to beer tested 5.2%, 8%, and 11% ABV over 60 days of wood maturation, finding that higher ethanol levels increased the extraction of wood-derived compounds. The same study also found that ethanol changed the microbial community by obstructing the growth of *Lactobacillus brevis* and *Gluconobacter oxydans*, while delaying fungal growth [35]. In the industrial barrel ageing of three beers, the higher-alcohol beers accumulated greater quantities of cis/trans oak lactone, eugenol and total polyphenols than the lower-alcohol beers. This study also showed that the outcome depends on the initial properties of the beer, not ethanol alone, as the samples clustered primarily by beer type and then by time during maturation [32]. Even when stored at 0 °C for three months, low-ABV lager still gained aldehydes, esters, higher alcohols, and wood-derived compounds. However, the effects were smaller than expected and were partly attributed to the beer’s low alcohol content [20]. Similarly, dark, sour, or high-extract beers may integrate intense wood notes more effectively than pale, delicate beers, for which even moderate extraction can significantly alter the sensory balance.

### 2.5. Reused Wood and Beer Style

Beer aged in wine and port barrels is affected not only by oak extraction but also by the transfer of compounds adsorbed from the previous beverage [1,34]. Therefore, reused wood can act as a carrier of both wood extractives and volatiles from previous beverages, imparting sensory traits to beer that are associated with wine, fortified wine, whisky, or other former contents [20,34]. This carryover can be measured. Oak chips that had been immersed in fortified wine released wood compounds and adsorbed wine volatiles into the beer, which had a greater impact on the beer’s composition than on wine or grape marc spirit under the same treatment [34]. The ethanol content of barrel-aged craft beers can increase due to the presence of residual fortified wine material, which contributes additional alcohol. This has been demonstrated in beers aged in port-conditioned barrels with lees present [36]. Port-conditioned oak has a particularly noticeable effect on stout. After six weeks, an imperial stout that had been matured with American oak chips that had been pre-soaked in Ruby Port exhibited higher concentrations of vanillic acid, ellagic acid, gallic acid, catechin derivatives, syringaldehyde and coniferaldehyde when the treatment involved repeated port conditioning. This system also increased the levels of ethyl esters, volatile phenols such as 4-ethylguaiacol and 4-ethylphenol, citral, and several sesquiterpenes [19].

Wine barrel maturation can also contribute to a significant increase in total polyphenols. Italian craft beers aged in wine barrels showed a total polyphenol content 2.5 times higher than that of other craft beers and 3.6 times higher than that of an industrial beer, with a similar antioxidant activity [37]. The content of polyphenols is determined by the characteristics of the barrels and their previous use. NMR metabolomics identified metabolites that distinguished the analysed beer samples, including 5-hydroxymethylfurfural, acetaldehyde, mannose and tryptophan [38]. Unless their sensory contribution was independently validated, these compounds should be interpreted as markers associated with sample differentiation rather than as compounds demonstrated to cause the observed flavor profile. In addition, the study found that wine barrels may affect the composition of beer differently depending on their previous use. For example, furfural and 5-methylfurfural were found to be significantly higher in beers aged in new barrels compared to those aged in barrels previously used to age red wines. This may be due to the fact that some of the extractives in the wood of wine barrels are depleted or transformed during the first use [32].

Beer style should be treated as an important element of process design. Dark, strong, sour or specialty beers may provide a more suitable matrix for intense vanilla, smoky, toasted and spicy notes. In pale or low-alcohol beers, contact with wood should be conducted more cautiously, because the lower intensity of the base profile may increase the perceptibility of wood-derived compounds. From a technological point of view, this means that wood species, degree of toasting, dose and contact time should be treated as interdependent parameters rather than independent process variables.

Table 3 presents a synthetic overview of the main parameters of wood application that are identified in the literature as factors determining extraction dynamics and product quality. This overview does not constitute a universal technological procedure but rather organizes the variables that require control during process design.

In summary, the effective use of wood requires the combined control of material properties and beer maturation conditions. Wood species and degree of toasting determine the potential composition of extracted compounds, whereas dose, contact time, temperature, alcohol content and beer style influence the dynamics and sensory effect of extraction. The lack of standardization of these parameters remains one of the main reasons for the limited comparability of research results and makes it difficult to directly transfer findings from the literature into brewing practice.

### 2.6. Microbiological Stability and Process Safety

Most microbiological studies on wood-aged beer concern barrels or experimental systems designed to reproduce barrel conditions. Accordingly, the evidence discussed in this section is distinguished between observations from full-scale barrel maturation, laboratory systems designed to reproduce barrel-associated microbial succession, and evidence directly applicable to fragmented wood where available (Table 4). Findings from barrel or model systems should not be interpreted as demonstrating equivalent microbial behaviour during short-term wood chip treatment. Bossaert et al. [11] matured conventionally fermented beer in oak and acacia barrels for 38 weeks. The microbial communities in both systems became dominated by *Pediococcus damnosus* and *Brettanomyces bruxellensis*. However, the oak-aged beer also contained a substantial proportion of *Acetobacter* spp., averaging 29.34% of the bacterial community, and a smaller fraction of *Paenibacillus* spp., averaging 2.74%. Both groups were almost absent from the acacia-aged beer. These findings demonstrate that wood species and barrel properties can influence microbial community structure, but they relate to prolonged barrel maturation rather than short-term chip treatment [11].

Temporal changes were also observed when three conventionally fermented beers differing in alcohol content and bitterness were matured in new and used oak barrels for 38 weeks [32]. Samples collected at the beginning and after 2, 12, and 38 weeks showed increases in bacterial and fungal population densities and substantial changes in community composition, beer chemistry, and sensory properties. These results demonstrate that the microbiological state of barrel-aged beer is dynamic and that measurements taken only at the end of maturation may not adequately describe the process [32].

The composition of the beer itself also determines which microorganisms can establish during wood maturation. In a controlled model containing four bacterial and four fungal species associated with barrel-aged beer, ethanol concentration and iso-α-acid level significantly influenced microbial community composition [35]. Higher ethanol levels inhibited the development of *Lactobacillus brevis* and *Gluconobacter oxydans* and delayed fungal growth, whereas iso-α-acids restricted *L. brevis* and promoted *Pichia membranifaciens*. A subsequent laboratory model based on a wooden disk and a defined eight-species community reproduced several microbial and chemical trends observed during industrial barrel maturation, confirming that wood, beer composition and microorganisms act as an interacting system [31].

The microbiological role of these organisms depends on the intended product. In sour and mixed-fermentation beers, *Brettanomyces*, lactic acid bacteria and, under controlled conditions, acetic acid bacteria may contribute to attenuation, acidification, ester formation and the development of phenolic or complex fermentation-derived attributes. In conventionally fermented beer intended to remain microbiologically stable, the same organisms may cause over-attenuation, excessive acidity, phenolic off-flavors, turbidity, over-carbonation, or package instability [31,32,35,39,40,41,42]. The classification of an organism as desirable or undesirable must therefore be linked to the intended beer category and process design.

Thermal, chemical, or physical preparation methods may reduce the microbial load of wood, but their influence on extractable compounds should also be considered. Procedures designed to improve microbiological safety may simultaneously alter concentrations of lactones, phenolic aldehydes, and volatile phenols. For this reason, sanitization cannot be evaluated independently of its effect on the chemical and sensory performance of the wood material [39].

## 3. Wood-Derived Compounds in Beer

### 3.1. Lactones

The lactones found in beer can be categorised into two main groups: oak lactones (which are derived from contact with wood) and aliphatic or furanone-like lactones (which are formed from raw materials or through oxidation and fermentation) (Table 5) [43,44]. The clearest sensory evidence of lactones in beer is for oak-derived lactones in barrel- or chip-aged products. Meanwhile, fruit-like γ- and δ-lactones often occur at low concentrations and appear to contribute mostly through mixture effects rather than individually [11,45].

Oak lactones are formed upon contact with oak barrels or chips and are not present in untreated controls [19]. They are chiefly formed by the acid-catalyzed lactonization of ring-opened 3-methyl-4-hydroxyoctanoic acid precursors, many of which enter beverages as glucosides or galloylglucosides that hydrolyze during maturation. As with wine, the final quantity of oak lactones in beer depends less on a single reaction step than on the full chain of precursor abundance, wood processing, extraction and time. Oak-treated beers contain both the cis- and trans-isomers of 3-methyl-4-octanolide, also known as whisky lactones. In fresh beers, oak lactones are absent, but during maturation in wooden barrels or contact with oak staves and chips, concentrations generally accumulate up to 250 µg/L [11,19]. Oak barrel ageing also links (Z)-oak lactone to maturation time and to woody and sweet odor notes in lager [33]. Whisky lactones were only detected in stout matured with port-soaked American oak chips in the oak-treated beers [19]. Oak barrels impart a greater quantity of lactones to conventionally fermented beer than acacia barrels do, demonstrating that the species of wood used controls the extent to which this process contributes to the final aroma of the beer [11].

Across various wood studies, American oak tends to produce higher levels of oak lactones than some European oak sources. This helps to explain the effects of the origin of the barrel on beer [46]. The time of maturation also matters. For example, three months with French oak cubes increased the levels of wood-derived compounds in lager, with heavily toasted cubes producing the highest levels of low-molecular-weight phenolics [20]. In a 300-day barrel ageing study, oak lactone behaved as a marker of flavor development over time [33].

Fermentation conditions can increase the levels of non-oak lactones. Pre-acidification with *L. gasseri* increased the levels of two 2(3H)-furanone lactones in boiled and fermented beer products, shifting the aroma towards sweeter and waxier notes with less green character [44]. Low pH also favors lactonization chemically, which is consistent with reviews linking lactone formation to acidic media and yeast-dependent pathways [43].

Fruit-like lactones appear to play a minor direct role in many beers, as measured γ- and δ-lactone levels in dry-hopped beers were far below the estimated threshold. Nevertheless, when combined with esters and oxygenated terpenes, they can enhance the overall fruity and peach-like aroma [45].

Not all lactones are desirable, however. Sotolon, a lactone that is absent from fresh beer but measurable at 5–42 μg/L after 6–24 months of ageing, is associated with a Madeira-like off-flavor in aged beer. Its formation appears to be linked to pro-oxidants, acetaldehyde and, possibly, acetoin and serine [47]. Representative oak-derived and non-oak lactones discussed in this section are shown in Figure 1.

### 3.2. Phenolic Compounds

Phenolic compounds in wood-matured beer originate from two complementary sources (Table 5). On the one hand, beer already contains a range of phenolic compounds derived from raw materials such as malt and hops, as well as from transformations that occur during the brewing process. On the other hand, maturation in wooden vessels introduces additional phenolics and modifies existing ones through mechanisms including the extraction of wood constituents, the degradation of lignin and hemicellulose, oxidation reactions, and other aging-related processes [14,18,48,49,50,51].

Understanding the role of phenolics in wood-aged beer therefore requires consideration of three closely related aspects. Firstly, it is important to examine the phenolic profile already present in the beer before it comes into contact with wood. Secondly, attention must be directed towards the mechanisms through which wood contributes new phenolic compounds or transforms those which are already present during maturation. Finally, a range of process variables—including wood species, barrel treatment, maturation time, and storage conditions—determine how many of these compounds are ultimately retained in the finished beer and, consequently, how they influence its sensory characteristics.

Prior to the occurrence of wood contact, the majority of beer phenolics are derived from barley malt, with smaller contributions from hops and further modifications during the processes of malting, mashing, boiling, and fermentation [48,49,50,51]. The process of brewing has been shown to effect changes in these compounds through a combination of enzymatic release and heat-driven reactions. Concurrently, losses occur through precipitation with proteins and adsorption to trub, yeast, and stabilizers [48].

In general, the main phenolic compounds found in beer are derivatives of benzoic and cinnamic acids, including ferulic, caffeic, vanillic, p-coumaric, gallic, protocatechuic, salicylic and syringic acids [52,53]. Over 50 phenolic compounds have been identified in beer, with 57 reported in a survey of craft beers, including 12 that had not previously been described in this context [53,54].

It has been demonstrated that contact with wood has the capacity to increase the total phenolic content and the concentration of specific wood-derived phenolic compounds in beer. However, the magnitude and direction of the effect are contingent on the type of wood and the processing method utilized. A repeated finding in studies of wood-matured beer is the increase in vanillic acid, syringic acid, gallic acid, catechins and lignin-derived aldehydes such as syringaldehyde and coniferaldehyde [14,19]. It is evident that levels of volatile phenols are subject to alteration during the process of barrel ageing, a phenomenon attributable to the pivotal roles played by wood extraction and microbial activity. For example, it has been observed that *Brettanomyces* catalyzes the conversion of hydroxycinnamic acids into volatile phenols during barrel ageing [2,55]. The use of French oak cubes and barrels increases the total phenolic content of lager beer, with the highest levels found in heavily toasted cubes and barrels [20]. Six weeks of maturation increased multiple phenolic compounds—including vanillic acid, ellagic acid, gallic acid, catechin, epicatechin, syringaldehyde and coniferyl aldehyde—in stout aged with Port-soaked American oak chips [19]. A broader review of wood-aged beverages concludes that even short maturation periods can significantly alter the composition of beer through extraction from toasted wood [2,9].

Phenolics are of significance in the realm of beer production due to their impact on characteristics such as flavor, color, haze, foam, and oxidative stability. This significance extends beyond their mere antioxidant capacity, as evidenced by studies conducted by Wannenmacher and Radonjić [48,56]. Beer phenolics have been demonstrated to be associated with antioxidant activity. Indeed, a positive correlation has been observed between hydroxycinnamic acids and 4-vinylguaiacol, on the one hand, and antioxidant measures in commercial beers, on the other [56,57]. Representative phenolic compounds relevant to the native beer matrix and to wood-associated maturation are summarized in Figure 2.

In the context of wood-aged beers, however, it has been observed that sensory gains do not always correspond directly with increases in phenolic compounds. Oak-treated stout exhibited discernible phenolic and volatile distinctions; however, a sensory analysis of variance (ANOVA) revealed no statistically significant differences among the treatments [19]. In a similar manner, wood-aged beer demonstrated significant antioxidant and cellular bioactivity in general. Nevertheless, the influence of wood was found to be comparatively negligible when considered in the context of the more pronounced effects associated with the utilization of dark malt [14]. Too much phenolic enrichment can also create tradeoffs. Very high polyphenol additions have been associated with haze, while lignan enrichment from wood chips or extracts has been linked to excessive bitterness and an unusual resin aroma [55].

### 3.3. Furanic Compounds

Furanic compounds constitute a chemically diverse group detected during beer production and maturation. Their presence can result from wood treatment, thermal reactions during brewing, ageing-related transformations, or compounds already present in the base beer [33,58,59,60,61,62,63]. Some furan derivatives have recognised odor properties associated with toasted, caramel-like, sweet, spicy, or other sensory notes [33,60,64]; however, analytical detection alone does not demonstrate a sensory contribution. Such attribution requires consideration of compound-specific concentration, sensory threshold, and (where available) odor activity or GC–O evidence [60,64].

From a chemical perspective, furfural and 5-hydroxymethylfurfural are classic products of biomass dehydration associated with lignocellulosic material, particularly cellulose and hemicellulose. This explains why toasted barrels are such plausible sources [59,65]. Similarly, the group of compounds discussed is formed in smoked malt. Pyrolysis of wood produces phenols, guaiacols, syringols, and some furan derivatives—compounds that may remain in the finished beer [62]. In barrel-aged lager, the levels of heterocyclic compounds increased in a sigmoidal pattern over 300 days. Several furan-related compounds rose alongside notes of roast, nuts, fruit, and sweetness [33]. The same study identified furfuryl ethyl ether as one of the heterocyclic compounds that increased during ageing, alongside ethyl 2-furoate. This finding reinforces the idea that furan chemistry is an integral part of the evolving barrel-aged aroma profile [33].

Similar conclusions are reached in beer-aging work outside the barrel context: furfural, 2-furfurylmethanol and, in particular, furfuryl ethyl ether can increase sharply during ageing, with furfuryl ethyl ether exceeding its beer flavor threshold and imparting a solvent-like note [60]. Furfuryl alcohol is important here because it is considered the main precursor of furfuryl ethyl ether in beer fingerprinting studies, linking routine brewing chemistry to the aromas produced during ageing [63].

Not all furans have the same sensory profile. In alcohol-free beer, for example, the 5-ethyl-3-hydroxy-4-methyl-2(5H)-furanone was one of the key odor-active compounds, contributing a curry-like, spicy, worty character. This shows that furan-derived molecules can be potent even when present in small amounts [64]. The structural diversity of the principal furan derivatives discussed in relation to beer production and maturation is illustrated in Figure 3.

### 3.4. Volatile Phenols and Spice Notes

Wood aging can produce clear increases in spicy and smoky notes in beer, and the strongest direct experiment found woody, vanilla-like, spicy, and smoky flavor notes rose after oak-chip aging alongside higher concentrations of guaiacol, 4-ethylguaiacol, eugenol, thymol, vanillin, and syringaldehyde [6]. Across barrel-aging studies and reviews, those spice-linked changes are attributed to extraction of wood-derived phenols and related compounds from the barrel matrix, with toasted wood specifically implicated in generating distinctive sensory profiles [9,18].

Volatile phenols in beer do not come only from wood, so wood-matured beer starts from a phenolic background already set by malt and yeast [14,47,66]. The main beer-relevant volatile phenols are 4-vinylguaiacol, 4-vinylphenol, 4-ethylguaiacol, and 4-ethylphenol, which have low sensory thresholds and are described as clove-like, spice, smoky, woody, leathery, and stable-like depending on concentration and mixture [66].

Wood adds another route: barrel aging releases volatile phenols, lactones, furans, and aldehydes that directly contribute toasted, spicy, and vanilla notes [9,33]. Toasting matters because it modulates the release of phenolic and volatile compounds, which is one reason different woods and toast levels produce different spice signatures [2,18]. Time changes the sensory picture. In barrel-aged beer, smoky, woody, whisky-like, and astringent notes increased until about 12 weeks and then declined later, while spicy and phenolic sensory scores dipped early and varied with wood species and time [11]. Over a longer 300-day lager aging, barrel contact introduced new coconut and vanillin notes and progressively intensified woody and caramel attributes, showing that phenolic spice sits inside a broader evolving aroma matrix rather than acting alone [33].

Barrel history also matters. In imperial stout aged with Port-soaked American oak chips, multiply used chips produced higher 4-ethylguaiacol, 4-ethylphenol, eugenol-linked oak character, and more vanilla/coconut tendencies than first-use simulation, even though sensory differences were descriptive rather than statistically significant [19]. By contrast, short 30-day maceration with low doses of oak or cherry chips produced little overall volatile change and no clear wood maceration effect on eugenol, suggesting that contact intensity can be too low to generate obvious spicy phenolic shifts [5]. Selected volatile phenols and related aroma-active compounds discussed in this section are shown in Figure 4.

### 3.5. Other Volatile Compounds Derived from Beer

Beyond volatile phenols, wood maturation shifts beer volatiles through both additive and subtractive aging; wood contributes lactones, aldehydes, furans, and other extractives, while the wood matrix can also adsorb native beer esters and terpenes [33,67]. The main pattern is not a single universal increase but a rebalancing of volatile classes that depends on time, wood species, barrel history, temperature, ethanol, and contact format [9,32,68].

Esters are the most consistently shifted non-wood class. In lager, ester levels increased across all treatments, with the largest increase occurring in barrel-matured beer by month three [20]. However, wood can also reduce native fruity and floral volatiles because linalool and 2-phenylethyl acetate decreased significantly with wood application, which is consistent with adsorption by the wood matrix [1,33,34]. Ethyl esters often shift during wood ageing, and barrel beers aged for shorter periods can retain a characteristic ester-rich profile [36,69]. Overall, higher alcohols increased in barrel-aged beers, with some, including 3-ethoxy-1-propanol and Z-3-hexenol, being absent from unaged controls [36].

Aldehydes also tend to increase during maturation. In oak-aged lager, for example, aldehydes peaked in the third month, reaching their highest levels in barrel-aged beer. It has been suggested that oxidation inside the barrel is a key driver of this process [20]. Longer ageing usually results in a broader volatile profile in beer. Untargeted volatilomics identified 162 differential metabolites across 12 classes over 300 days of barrel ageing, and volatile fingerprinting placed the beer from the longest-aged barrel in the cluster with the highest number of volatiles [33,36]. Examples of non-wood volatile compounds whose concentrations may change during wood or barrel maturation are presented in Figure 5.

## 4. The Impact of Wood Chips on Beer Quality

The effects of wood chips on beer quality can be attributed only when the treated beer is compared with a control from the same production batch and stored under equivalent conditions. Beer continues to change during storage; therefore, an untreated variant is required to separate storage-related transformations from those caused by wood contact. A barrel-aged variant is a second, mechanistically different reference because a barrel combines extraction with oxygen transfer through its structure, effects of barrel age and previous contents and, in selected sour or mixed-fermentation products, microbial succession [5,6,7,8,11,13,14,19,20,31,32,33,35,39,40,41,42,67]. These variants represent distinct maturation environments rather than successive levels of product quality.

### 4.1. Physicochemical and Sensory Effects

Direct beer studies demonstrate that the magnitude and direction of changes induced by wood chips are conditional. Short maceration with cherry or oak chips at 1 g/L produced only limited, beer-dependent changes in volatile composition [5]. In porter, American and French oak chips applied at 3–7 g/L during fermentation or maturation affected fermentation efficiency, titratable acidity and the volatile profile, but the relationships between dose, extract and alcohol concentration were not monotonic [8]. In pale and dark beers treated with 2 g/L of oak chips, American oak increased selected phenolic acids relative to French oak; however, malt type explained more of the overall variation in phenolic composition and antioxidant activity than wood origin [14]. Consequently, no universal dose–response relationship can be inferred, and the initial beer matrix remains a major source of variation.

Chemical and sensory responses are related but not interchangeable. Oak chip treatment increased woody, vanilla-like, spicy and smoky attributes together with the concentrations of nine monophenols, including vanillin, syringaldehyde, guaiacol and eugenol [6]. Model beer solutions subsequently showed that extraction was modified by the chip dose and properties of the liquid phase [7]. Conversely, lager matured at 0 °C with oak cubes or in a barrel showed increased concentrations of several phenolic and volatile compounds, whereas an untrained acceptance panel detected no significant differences among the treatments [20]. An increase in wood-derived compounds must therefore be interpreted as a compositional change, not as evidence of sensory improvement. Quality improvement requires that the introduced attributes remain coherent with the base beer and do not produce excessive astringency, bitterness, or a drying aftertaste. The experimental designs, principal findings, and comparative relevance of the studies discussed in this subsection are summarized in Table 6.

### 4.2. Comparative Interpretation and Methodological Limitations

Wood chips and barrels share the migration of extractable phenolic and volatile constituents from wood into beer, but only part of the barrel maturation mechanism is reproduced by dispersed wood fragments. Their high surface-area-to-volume ratio facilitates extraction and permits the material dose and contact time to be defined directly [5,6,7,8]. Conditioned chips can also transfer compounds retained from a previous alcoholic beverage [19,34]. They do not, however, reproduce the spatial heterogeneity, gradual depletion of extractives or long-term oxidative and microbiological interactions of a whole barrel. Results from barrel studies are therefore useful for defining the comparator, but they cannot be interpreted as direct evidence of the effects of wood chips [11,13,14,19,20,31,32,33,35,39,40,41,42,67].

The evidence base is further limited by the small number of experiments containing an untreated control, wood fragments and a barrel produced from the same beer. The study by Wyler et al. [20] provides the closest comparative design, but it concerns oak cubes rather than chips, lager matured at 0 °C, and sensory acceptance evaluated by an untrained panel. Moreover, the treatment variants differed in container material and volume, so wood format was not the only experimental factor that varied. The absence of a statistically significant difference in sensory acceptance should therefore not be interpreted as evidence of sensory equivalence. Demonstrating equivalence would require an experimental design and statistical analysis specifically intended for equivalence or non-inferiority testing. Comparisons between separate studies are affected by differences in beer style, alcohol concentration, wood form, effective contact area, maturation time, and storage temperature. A chip dose expressed in g/L is not directly equivalent to the wood surface available in a barrel. Conclusions should therefore distinguish direct beer–chip evidence from model system results and indirect evidence derived from barrel maturation.

Overall, wood chips are a controllable material for obtaining defined extractive and sensory effects, but they are not a complete technological equivalent of a barrel. Their effectiveness should be demonstrated against a matched untreated control through concordant physicochemical and sensory results; a statistically significant analytical change alone is insufficient to establish improved beer quality.

## 5. Beer Styles and Product Categories Shaped by Barrel Maturation

Barrel maturation is used mainly in beer categories in which the base beer has sufficient sensory intensity, alcohol content or microbiological complexity to integrate wood-derived characteristics. In most cases, barrel ageing does not create a separate beer style in itself but modifies an existing beer matrix by introducing additional aromatic, flavor, and structural attributes. These changes may include notes associated with wood, vanilla, coconut, toast, smoke, spice and controlled oxidative or microbiological development [1,2,6,7,11,12]. Therefore, the technological role of barrel maturation depends not only on the wood and the barrel environment but also on the composition and sensory potential of the base beer.

The main beer categories associated with barrel maturation are summarized in Table 7 and Table 8. This classification should not be treated as a complete style taxonomy. Its purpose is to organize the main beer matrices discussed in the literature in relation to barrel maturation, wood-derived compounds, and microbiological changes.

### 5.1. Strong, Dark, and High-Alcohol Beers

Strong and dark beers are frequently represented in studies of wood and barrel maturation. Their relatively intense malt-derived sensory profiles and, in some cases, higher alcohol concentrations may modify the perception and extraction of wood-derived compounds; however, suitability for barrel maturation remains product- and process-specific [2,11,32]. Of these styles, Imperial Stout is most closely associated with barrel ageing, particularly in ex-bourbon casks. Its rich profile of roasted malt, chocolate, coffee and dark fruit integrates naturally with the vanilla, caramel, and charred oak notes extracted from bourbon barrels [67]. The beer’s dense body and high alcohol content are conducive to extended maturation, which can extend over several months or even years in wooden barrels [1,11,32,35]. Residual bourbon has been demonstrated to increase the final alcohol level, while alternative barrels, such as those utilized for rum, tequila, or cognac, have been shown to introduce additional layers of flavor reminiscent of tropical fruits, agave, minerals, or brandy [1,20,34].

Barleywine and related strong ale categories are occasionally associated with extended storage or barrel maturation [38,70]. However, the reviewed evidence for these individual styles is less direct and systematic than that available for stout-type beers, barrel-aged lagers and sour or mixed-microbiota beers [19,20,32,33]. These categories should therefore be regarded primarily as examples of beer matrices in which wood or barrel maturation may be applied rather than as evidence supporting general technological recommendations concerning wood maturation. Where specific barrel types or sensory effects are discussed, they should be linked only to studies directly investigating the corresponding beer style.

Strong Belgian-style ales may also be subjected to wood or barrel maturation, although direct comparative evidence for these beer categories remains limited. In such applications, the technological role of the barrel should primarily be interpreted in terms of wood-derived extraction and, where relevant, transfer of compounds from previous barrel contents. Evidence concerning resident *Brettanomyces*, lactic acid bacteria, and long-term microbial succession is substantially stronger for Lambic and Flemish sour-beer production and is therefore discussed separately in Section 5.2 [71,72,73,74].

Other strong specialty beers, including wheat wine, have also been associated with barrel maturation, although the direct experimental evidence for style-specific technological effects remains limited [9,38,67,75].

### 5.2. Sour and Mixed-Microbiota Beers

Sour beers and beers produced with the participation of mixed or non-standard microbiota represent another important group associated with barrel maturation. In these beers, the barrel is not only a source of wood-derived compounds but also an environment in which microbial communities may influence the chemical and sensory development of the product [31,32,35,39,40,41,42]. Studies on sour beer production via barrel ageing indicate that changes in microbial community composition are linked with changes in beer chemistry during maturation [32]. The activity of microorganisms such as *Brettanomyces* yeasts, lactic acid bacteria, and acetic acid bacteria may contribute to acidity, phenolic notes, ester formation, and other fermentation-derived attributes, but it may also increase the risk of instability and sensory defects [31,32,35,39,40,41,42]. For this reason, this category requires particular attention to microbiological control and barrel sanitation [39,40].

The wood maturation process is equally important for both foeder-aged dark beers and sour hybrid beers, which are inspired by traditional Flemish brewing methods. These beers combine the richness of dark malt with acidity, fruitiness, and a ‘funky’ flavor. Due to their much lower surface-area-to-volume ratio, foeders allow for more restrained direct wood extraction than small barrels. The primary contribution instead comes from the res-ident populations of *Lactobacillus*, *Pediococcus,* and *Brettanomyces* that inhabit the wood and influence successive generations of beer. The result is a complex balance of malt sweet-ness, acidity, and character derived from microbes [72,73,76,77].

Lambic represents the archetype of wood-matured sour beer. Produced through spontaneous fermentation using unmalted wheat and aged hops, lambic is traditionally matured in old oak barrels or large wooden foeders for up to one to three years [71,72,78,79,80,81]. The process begins with environmental inoculation during cooling and continues through fermentation and maturation in wooden vessels, whose inner surfaces can also act as an additional inoculation source [71,78,79,82]. Rather than serving mainly to add strong oak character, these barrels help establish a stable microbial ecosystem and provide a microaerobic environment that supports the desired fermentation course [32,78]. The microbial community includes wild oxidative yeasts early on, *Saccharomyces* during main fermentation, lactic acid bacteria such as *Pediococcus* and sometimes *Lactobacillus* during acidification, acetic acid bacteria such as *Acetobacter*, and later *Brettanomyces*/*Dekkera* during maturation [80,82,83,84,85]. Across this long maturation, a characteristic microbial succession gradually acidifies and attenuates the beer, with *Pediococcus* driving lactic acid production, acetic acid bacteria contributing acetic acid and acetoin, and *Brettanomyces* contributing late-stage attenuation and characteristic flavor development [78,80,85,86]. This is what gives lambic its acidity and complex “funk”-associated profile, including *Brettanomyces*-linked volatile phenols and other distinctive aromatic compounds [86,87,88].

Gueuze builds directly upon the lambic tradition and demonstrates the importance of wood maturation through the art of blending, because lambic beers are produced by spontaneous fermentation and maturation in wooden barrels and are then used as the base for lambic-derived beers such as gueuze [32,79,83]. This classic Belgian style is produced by combining young and old lambics, typically one-, two-, and three-year-old beers, which have developed distinct microbial and metabolite profiles during barrel maturation [83,89,90,91,92]. Blending is used to manage batch and barrel variability that arises from spontaneous inoculation, differences among wooden barrels, and shifts in temperature, pH, dissolved oxygen, and microbial composition during long maturation [78,80,83]. That variability means gueuze complexity reflects not only blending skill but also natural differences among individual casks over time, and even bottled gueuze shows considerable bottle-to-bottle variation in metabolite profiles [78,83]. The balance of acidity, funk, dryness, and carbonation comes from both the chemistry of aged lambic and the bottle refermentation of the blend: gueuze is more carbonated than lambic, while lambic and gueuze are characterized by lactic acid, acetic acid, ethyl lactate, ethyl acetate, and *Brettanomyces*-associated phenols such as 4-ethylphenol and 4-ethylguaiacol [32,83,86,92,93].

Another notable example of the wood-aged sour beer is the Flanders Red Ale, which has rich vinous notes, sweet cherry fruitiness, and balsamic acidity. First of all, it is aged in large oak foeders for at least 18 months, during which time the beer acquires a wine character from the tannins extracted from the wood. Secondly, oxygen penetrates the wood of the foeders, allowing the development of a red wine flavor, as well as balsamic notes due to the formation of acetic acid by wild yeast and bacteria. However, in Flanders Red Ale, the beer from the foeders is merged with young beer to give the finished product a less persistent acidity and a more pleasant aroma [40,73,74,76].

Flanders Oud Bruin, also known as Old Brown Ale, has some similarities with Flanders Red, but tends to have a sweeter, maltier flavor with notes of dark fruit and milder acidity. The role of wood maturation varies more in this style. Some examples are aged in foeders or barrels, where mild oxidation and tannin extraction contribute to complexity, while others rely less heavily on wood. As with Flanders Red, blending is often used to fine-tune the balance between sweetness and sourness [40,73,74,76].

Modern American Wild Ales and barrel-aged sour beers have expanded the possibilities of wood maturation even further. Brewers frequently use previously filled wine barrels, both red and white, as vessels for mixed fermentation involving *Brettanomyces*, *Lactobacillus*, and *Pediococcus* cultures. Residual wine character can contribute tannins, fruit notes, and oxidative complexity, while the barrel itself serves as a habitat for microbial activity. Fruit additions such as cherries, peaches, raspberries, and other fruits are commonly introduced during barrel aging, creating intricate interactions between fruit sugars, microorganisms, and wood-derived compounds. The result is a highly experimental category in which barrel selection, microbial cultures, and fruit choices vary widely among producers [89,94,95,96].

In contrast, traditional Berliner Weisse and Gose historically have little association with wood. These light, tart, and relatively low-alcohol styles are commonly produced through rapid lactic fermentation or kettle souring in stainless steel vessels. However, some modern craft brewers have experimented with barrel-aged interpretations, using neutral barrels to introduce subtle oxidative or funky characteristics while preserving the beers’ refreshing and delicate nature. In these cases, wood maturation represents innovation rather than adherence to historical practice [11,32,39,97,98,99].

The classical solera system originates from wine production and involves fractional withdrawal and replenishment over successive ageing cycles [100,101]. Although this process provides a useful technological analogy for sequential blending and long-term maturation, evidence derived from Sherry or other wine systems should not be treated as direct evidence for beer. Dedicated studies characterizing the chemical, sensory and microbiological evolution of beer under solera-type maturation remain limited.

### 5.3. Other Beer Styles

Barrel-aged lagers form a separate product category that is relevant mainly from the perspective of process control and aroma development. Studies comparing barrel-aged lager beer with unaged beer have shown that barrel maturation can modify the volatile and metabolic profile of lager beer [13,33]. Compared with strong dark or sour beers, lager-type beers usually have a more delicate sensory matrix, which means that wood-derived notes may become perceptible more easily and may disturb the balance of the product if maturation is not properly controlled. Therefore, in this category, barrel maturation should be conducted with particular attention to contact time, wood impact and the compatibility of barrel-derived notes with the base beer profile [13,33].

Specialty and craft beers constitute a broader group in which barrel maturation is used primarily as a tool for product differentiation and sensory complexity. Studies on diverse-aged craft beers indicate that maturation conditions can differentiate the volatile profile of beer [36]. In addition, the development of tools such as the barrel-aged beer wheel confirms the need for structured sensory assessment of beers matured in barrels, especially because their profiles may include a wide range of descriptors related to wood, oxidation, fermentation and microbial activity [12]. In this group, barrel maturation should be understood as a technological and sensory tool rather than as a uniform style-defining procedure.

These categories show that the effect of barrel maturation depends strongly on the properties of the base beer. Strong, dark and high-alcohol beers may better integrate intensive wood-derived notes, while lager-type beers require more restrained maturation conditions [13,19,33,67]. Sour beers and beers with complex microbiota require a different technological approach, because their final quality depends not only on extraction from wood, but also on microbial succession and chemical changes during ageing [31,32,35,39,40,41,42]. This distinction is important when considering wood chips as an alternative to barrels, because the suitability of wood-derived extraction depends not only on the form of wood but also on the beer matrix into which these compounds are introduced [5,6,7,8,11].

## 6. Operational, Economic, and Environmental Implications and Research Priorities

From a brewery perspective, wood chips and barrels constitute different maturation systems rather than directly interchangeable sources of wood flavor. The available evidence supports greater control of wood dose and contact time when fragmented wood is used, but it does not demonstrate that this approach is universally less expensive or environmentally preferable. Direct brewery-scale comparisons remain scarce, and much of the evidence concerning accelerated maturation, barrel regeneration, and resource use originates from wine, spirits or model alcoholic matrices [21,102,103,104,105,106,107,108,109,110,111]. These studies identify relevant mechanisms and cost drivers, but their results cannot be transferred quantitatively to beer because the ethanol concentration, pH, colloidal composition, microbiological conditions and required sensory endpoints differ among beverages [1,2,10]. The following assessment therefore concerns operational consequences and variables requiring measurement rather than a general economic ranking of the two technologies.

### 6.1. Operational Implementation and Process Control

Wood chips can be introduced into stainless-steel maturation vessels, allowing wood species, degree of toasting, dose and contact time to be defined as separate process parameters [5,6,7,8]. Their high surface-area-to-volume ratio promotes mass transfer and may enable the treatment to be stopped when a predefined analytical or sensory endpoint is reached. The reported contact periods nevertheless vary substantially. Measurable changes have been observed after 21 days with 2 g/L of oak chips [14], after 30 days with 1 g/L of oak or cherry chips [5], and after 2 or 3 months with oak chips or cubes [8,20]. By comparison, barrel studies have covered 38 weeks [11,32] and up to 300 days [13,33]. These durations are not directly comparable because the experiments differed in terms of their beer matrix, wood form, temperature, alcohol content, and target product profile. They show that fragmented wood can support a shorter and more readily monitored extraction stage, but they do not establish a universal processing time or sensory equivalence with barrel maturation.

Industrial implementation requires a raw material specification and a process control plan rather than dosing based only on wood mass. The specification should define botanical identity and origin, particle-size and effective-surface distributions, moisture content, degree and homogeneity of toasting, microbiological status, packaging and storage conditions. The validated operating procedure should additionally cover any chemical or thermal preparation, containment and removal of the material, stage of addition, dose, temperature, contact time and release criteria based on analytical, sensory, and microbiological results. From a process control perspective, the addition of dry porous wood to finished beer may introduce entrained air and therefore represents a potential source of oxygen exposure. However, the magnitude of this effect has not been sufficiently quantified for wood chip addition under brewery conditions. In oxygen-sensitive products, measures intended to minimise air entrainment or the use of inert gas handling may therefore be considered, but their effectiveness should be validated for the specific process. Equal doses expressed in g/L may provide different extraction surfaces and chemical profiles because wood anatomy, seasoning, and thermal treatment vary between batches. Supplier documentation should therefore be supplemented by verification of incoming material and monitoring of the treated beer.

Barrel processes are affected by vessel geometry, stave thickness, toasting heterogeneity, barrel age, previous contents, depletion of extractives, and retained microbiota [11,13,19,31,32,33,34,35,67,104,112]. These factors make a common endpoint more difficult to define and increase variation between vessels. Sanitation also differs between the systems. A stainless steel tank can be cleaned using standard brewery procedures, whereas the microbiological status of chips must be controlled before addition. In barrels, microorganisms can persist within porous wood and may either support the intended development of sour or mixed-fermentation beer or constitute a contamination source for products requiring microbiological stability [39,71]. Reuse introduces an additional source of variation: successive use of chips reduces their extractive capacity [34,110], while older barrels may shift from an extraction-dominated function towards oxygen transfer or microbiological maturation. Regenerated barrels and replacement inserts can extend vessel life [104,106,111], but they alter the effective wood surface and should be validated as distinct technological variants.

### 6.2. Cost Structure and Resource Utilization

The following expression is proposed as a conceptual process-based accounting framework rather than as a validated economic model. Its purpose is to identify mutually exclusive cost categories that should be parameterized using brewery-specific data over a clearly defined assessment period and allocated to the volume of beer meeting release specifications.

A conceptual process-based economic model should include at least

$$C_{\mathrm{unit}}\frac{C_{\mathrm{material}}+C_{\mathrm{equipment}}+C_{\mathrm{space}}+C_{\mathrm{labour}}+C_{\mathrm{sanitation}}+C_{\mathrm{utilities}}+C_{\mathrm{quality}}+C_{\mathrm{loss}}+C_{\mathrm{inventory}}-R_{\mathrm{residual}}}{V_{\mathrm{released}}}$$

where *C_material_* represents consumable wood material such as chips, cubes or replacement inserts; *C_equipment_* represents depreciation, leasing or maintenance costs allocated to maturation vessels and related equipment; *C_space_* represents production and storage space; *C_labour_* covers preparation, transfer, sampling, handling and removal operations; *C_sanitation_* covers dedicated cleaning and sanitation inputs; *C_utilities_* covers energy, cooling, water and compressed gases not already assigned to sanitation; *C_quality_* includes chemical, microbiological and sensory verification; *C_loss_* represents beer losses through retention, evaporation, leakage, sampling or rejection; *C_inventory_* represents the cost of capital tied up during maturation; *R_residual_* represents recoverable value from reuse, resale or alternative utilisation; and *V_released_* is the volume of beer meeting release specifications. The unit cost should subsequently be calculated relative to the volume of beer that meets release specifications, rather than the volume introduced into the maturation system.

To avoid double counting, the purchase cost of a complete barrel should be allocated through *C_equipment_* rather than simultaneously included in *C_material_*, whereas replaceable wood inserts or other consumable wood elements may be assigned to *C_material_*. Sanitation inputs and utilities should likewise be assigned to only one category. The assessment period, number of batches or maturation cycles, depreciation method and treatment of residual value should be stated explicitly.

Wood fragments are generally described as less expensive to purchase than complete barrels (Table 9), partly because smaller pieces of wood that cannot be used in cooperage can be processed into chips or other maturation products [104]. However, a lower material price does not automatically result in a lower cost per liter. Additional expenditure may arise from quality verification, microbiological preparation, containment of the wood inside the tank, removal after maturation, beer losses retained in the material, and possible filtration requirements.

Barrels involve higher capital expenditure and require dedicated storage capacity, handling, and periodic maintenance. Their long process duration also increases inventory levels and the period during which capital is tied up in the product. Additionally, barrel ageing may involve losses through evaporation, absorption by wood, leakage and the removal of non-saleable residues. Although these losses are recognised in alcoholic beverage production, their magnitude has not been quantified sufficiently for different categories of barrel-aged beer [103,104].

Barrel service life is another economically relevant variable. Extractable compounds are depleted during repeated use, but the barrel may continue to function as a vessel for oxidative or microbiological maturation. Various systems based on inserts, replacement wooden components or the regeneration of exhausted barrels have been investigated in wine production [106,111]. Such methods may extend barrel service life, although their economic and technological suitability for brewing requires separate validation.

### 6.3. Sanitation, Reuse and End-of-Life Management

Sanitation requirements differ fundamentally between removable wood chips and complete wooden barrels. A stainless steel tank can be cleaned using standard brewery procedures, whereas the wood material must be controlled before addition. Relevant measures include verified supplier hygiene, appropriate packaging, controlled storage and a validated preparation method. The selected treatment must reduce microbiological risk without causing uncontrolled changes in the extractable fraction of the wood.

Barrel sanitation is more complex because microorganisms can penetrate and persist within the porous wood structure. The internal surfaces of barrels may act as sources of microbial inoculation, particularly during the production of lambic and other beers based on mixed microbiota [39,71]. This feature may be technologically desirable in traditional sour beer production, but it constitutes a contamination risk where microbiological stability is required.

Methods investigated for barrel sanitation include hot water, steam, sulfur dioxide, ozone, ultrasound and other physical or chemical procedures [39]. More recent research has examined plasma-activated water as a method for barrel disinfection [109]. No single procedure can be assumed to ensure complete sanitation under all conditions because efficacy depends on the microorganism, treatment intensity, penetration depth, barrel condition and presence of deposits. Sanitation methods may also affect barrel integrity and the composition of subsequently extracted compounds.

Reuse affects both economic efficiency and product repeatability. Oak chips can be reused, but their capacity to release volatile and phenolic compounds decreases as the readily extractable fraction is depleted [34,110]. The sensory effect of a reused material may therefore differ substantially from that of first-use chips. Reuse should not be defined only by the number of cycles; it should be evaluated through the residual concentrations of relevant compounds, sensory performance and microbiological safety.

Barrels can be reused for numerous maturation cycles, although their function changes over time. New barrels primarily provide strong extraction, whereas older barrels may serve mainly as vessels for oxygen transfer or microbial maturation. Regeneration using internal wooden elements or other systems can partially restore the availability of extractable compounds and extend vessel life [106,111]. However, such interventions alter the effective wood surface and should be treated as a new technological variant rather than as simple continuation of the original barrel process.

Spent chips, damaged barrels and cooperage by-products may constitute secondary biomass streams. Potential pathways include energy recovery, bio-based materials, extraction of residual compounds or other forms of industrial valorization [104,113,114,115,116,117,118]. The broader brewing sector has developed numerous circular-economy strategies for the valorization of spent grain, yeast, hops and other processing residues [113,114,115,116,118,119]. Nevertheless, the environmental benefit of spent wood management should not be assumed solely because the material is biodegradable. Transport, drying, thermal treatment, contamination with beer or cleaning agents, and the energy required for further processing must also be included.

### 6.4. Product Value and Consumer Acceptance

Production cost, sensory quality, retail price, and consumer willingness to pay are separate variables. An analytically measurable increase in wood-derived compounds does not necessarily produce higher sensory acceptance or greater market value. Similarly, a higher retail price for barrel-aged beer may reflect scarcity, brand positioning, packaging, production narrative, or perceived craftsmanship rather than only the cost of maturation.

The market assessment of wood-treated beer should therefore distinguish between the following factors:

1. the instrumental effect of wood on chemical composition;
2. the sensory quality evaluated by a trained panel;
3. consumer acceptance;
4. willingness to pay;
5. intended market positioning;
6. the profitability of the product after accounting for production and distribution costs.

General beer consumer studies indicate that product knowledge, involvement, and information provided during evaluation can modify consumer responses and acceptance [4,120,121]. However, these findings do not demonstrate that experienced consumers specifically prefer woody, smoky, vanilla, sour or phenolic attributes in wood-aged beer. Such preferences should therefore be treated as hypotheses requiring dedicated consumer studies of wood-treated and barrel-aged beers.

Future consumer studies should therefore combine blind and informed evaluation. Blind testing can determine the sensory acceptance of the product itself, whereas informed testing can assess the influence of information concerning barrel ageing, wood species, craft production, sustainability or previous barrel contents. Price sensitivity and willingness to pay should be measured independently from overall liking.

### 6.5. Environmental Assessment

Potential environmental advantages of wood chips include a shorter maturation period, reduced demand for dedicated barrel storage and the possibility of using small wood fractions or cooperage by-products. However, these advantages have not been demonstrated through comparative life-cycle assessment for beer production.

A complete environmental evaluation should include the following elements:

- forestry origin and certification of the wood;
- transport distance;
- seasoning, drying, and toasting energy;
- manufacture of barrels or wood fragments;
- packaging and transport of chips;
- water, heat, and chemicals used for sanitation;
- refrigeration and storage energy during maturation;
- product losses;
- number of reuse cycles;
- end-of-life treatment;
- environmental burden associated with replacement materials and process equipment.

The environmental comparison should be performed using a clearly defined functional unit, such as 1 L of packaged beer meeting specified quality requirements. The functional unit alone, however, is insufficient unless the system boundary is also defined. Beer-specific LCA has previously used 1 L of beer as a functional unit within a cradle-to-grave system boundary [122]. Depending on the objective of the assessment, the comparison may adopt a gate-to-gate brewery boundary, a cradle-to-gate approach including wood production and transport, or a cradle-to-grave approach additionally accounting for packaging, distribution, and end-of-life processes. The same system boundary should be applied to wood chip and barrel scenarios, while allocation rules should explicitly address barrel service life, reuse cycles, regenerated components, product losses and secondary uses of spent wood. Comparing only the mass of wood used would disregard differences in maturation duration, beer losses, vessel service life and energy consumption. Existing beer life-cycle studies provide a methodological basis for defining functional units and system boundaries, but they do not resolve the specific comparison between wood chips and barrels [104,113,114,115,116,117,118,119].

### 6.6. Research Priorities

The literature indicates several areas in which additional evidence is required.

First, comparative experiments should include beer without wood contact, beer matured with wood chips and barrel-aged beer produced from the same base batch. Wood species, degree of toasting, effective contact area, temperature, oxygen exposure, and maturation time should be reported in sufficient detail to distinguish extraction effects from barrel-specific transformations.

Second, process parameters require standardization. Future studies should report chip size distribution, dose in g/L, wood surface area where possible, treatment method, stage of addition, temperature, contact time, beer alcohol content, pH, bitterness, and initial phenolic and volatile composition. Replication should include independent production batches rather than analytical replicates alone.

Third, brewery-scale techno-economic assessments are needed. These should apply process mapping, activity-based costing, and sensitivity analysis. At a minimum, the analyses should quantify material cost, vessel utilization, labour, maturation time, inventory cost, sanitation, utilities, product losses, quality control expenditure, rejection risk, and residual value of the wood.

Fourth, microbiological studies should focus directly on wood chips rather than relying primarily on barrel-derived evidence. Research should determine the microbial load of commercially available materials, the effectiveness of preparation methods, and the influence of treatment on the chemical and sensory properties of the chips.

Fifth, the reuse of wood should be evaluated over consecutive cycles. Such studies should quantify the depletion of phenolic and volatile compounds, changes in sensory performance, accumulation of beer-derived constituents, and microbiological risk [34,107,110,111].

Sixth, environmental research should compare wood chips and barrels using life-cycle assessment. The analysis should include the origin of the wood, energy used for processing, maturation duration, storage requirements, number of use cycles, product losses, and final waste management route.

Finally, consumer studies should integrate instrumental analysis, descriptive sensory evaluation, acceptance testing and willingness-to-pay measurements. This would determine whether analytically detectable differences provide a market benefit sufficient to offset the additional cost and operational complexity of wood maturation.

Overall, wood chips provide a potentially flexible means of introducing selected wood-derived characteristics within standard brewery infrastructure. Their advantages relate primarily to process controllability and the possibility of reducing maturation time. However, their economic and environmental superiority over barrels has not yet been demonstrated quantitatively. Establishing such superiority requires comparative research conducted at pilot or industrial scale using clearly defined quality specifications and a complete account of operational costs, product losses, and resource consumption.

## 7. Conclusions

Wood chips are a useful technological tool for modifying the chemical and sensory profile of beer through the extraction of selected wood-derived compounds. Their relatively high effective contact area can accelerate extraction compared with the gradual extraction characteristic of whole wooden vessels. However, the available evidence does not demonstrate that wood chips reproduce an equivalent finished product in a universally shorter maturation time. Wood chips are therefore not a complete technological equivalent of barrels, which additionally provide a distinct environment for oxygen transfer, long-term chemical transformation, carryover from previous contents and, in selected beer categories, microbial succession.

However, wood chips are not a full equivalent of barrels. Their effect is primarily extractive, whereas a barrel creates a more complex maturation environment involving micro-oxygenation, long-term chemical transformations and the potential participation of microorganisms. Barrel maturation is particularly relevant for strong, dark, sour, mixed-fermentation and specialty beers, where the base matrix and/or microbial activity allow wood-derived compounds to be integrated into a more complex sensory profile.

The effectiveness of wood chip application depends on the combined control of wood species, degree of toasting, dose, contact time, temperature, alcohol content and beer style. The main risks include excessive extraction, dominance of woody notes, increased astringency, variability between batches and the possibility of undesirable microbiological changes.

Further research should focus on the standardization of process parameters, the comparison of different wood species under the same technological conditions, the assessment of microbiological stability, and the quantitative analysis of the economic and environmental efficiency of using wood chips in breweries. Equivalence between chips and barrels in sensory quality, microbiological development, total production cost, and environmental performance remains unproven and requires matched pilot- or brewery-scale comparisons.

## Figures and Tables

**Figure 1:**
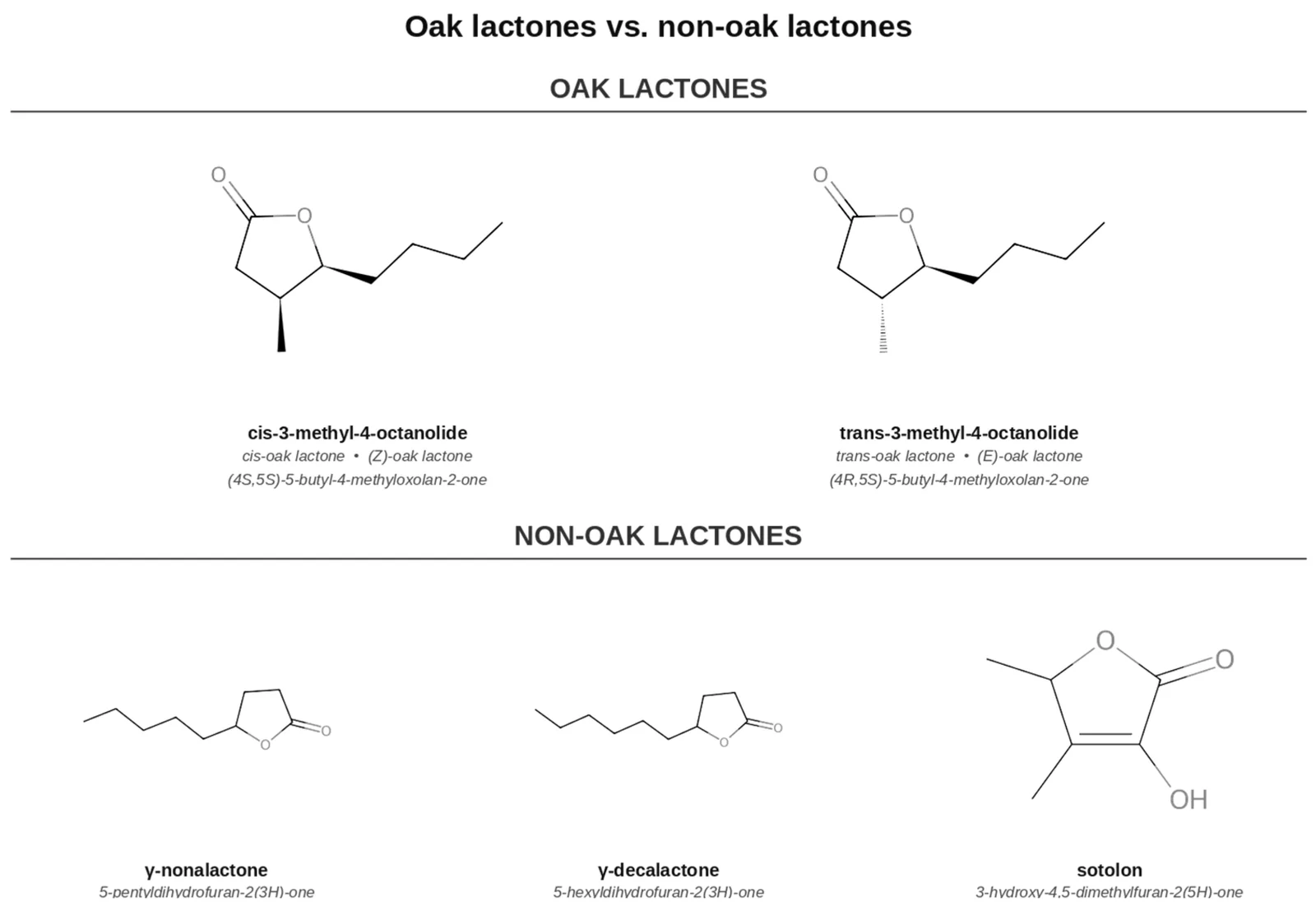
Selected lactones relevant to beer maturation, including cis- and trans-oak lactones and representative non-oak lactones discussed in relation to beer aroma and ageing [11,19,33,43,44,45,47]. Chemical structures prepared by the authors.

**Figure 2:**
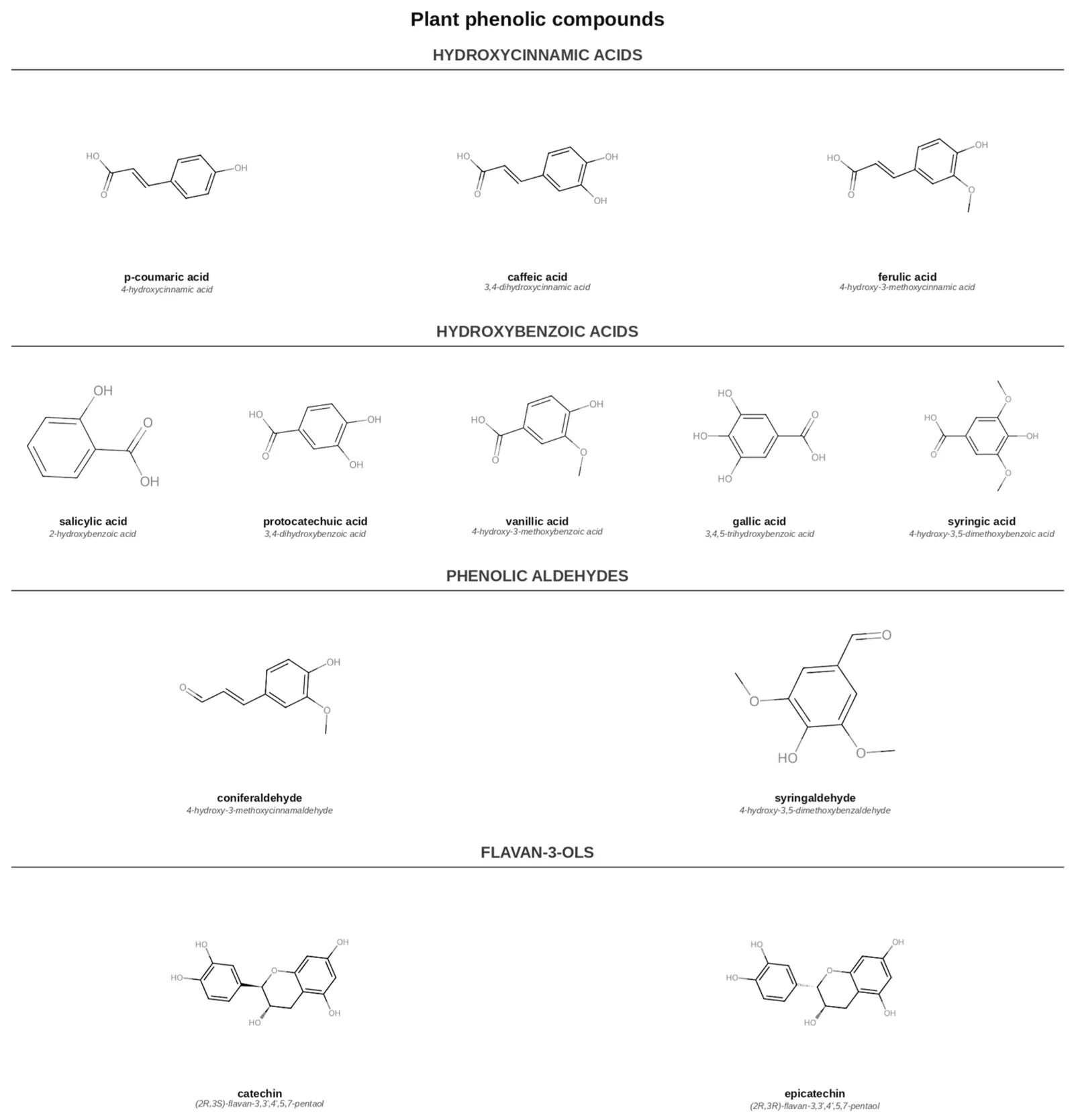
Selected phenolic compounds relevant to beer and wood-associated maturation, grouped as hydroxycinnamic acids, hydroxybenzoic acids, phenolic aldehydes and flavan-3-ols [14,18,48,49,50,52,53,54,56,57]. Chemical structures prepared by the authors.

**Figure 3:**
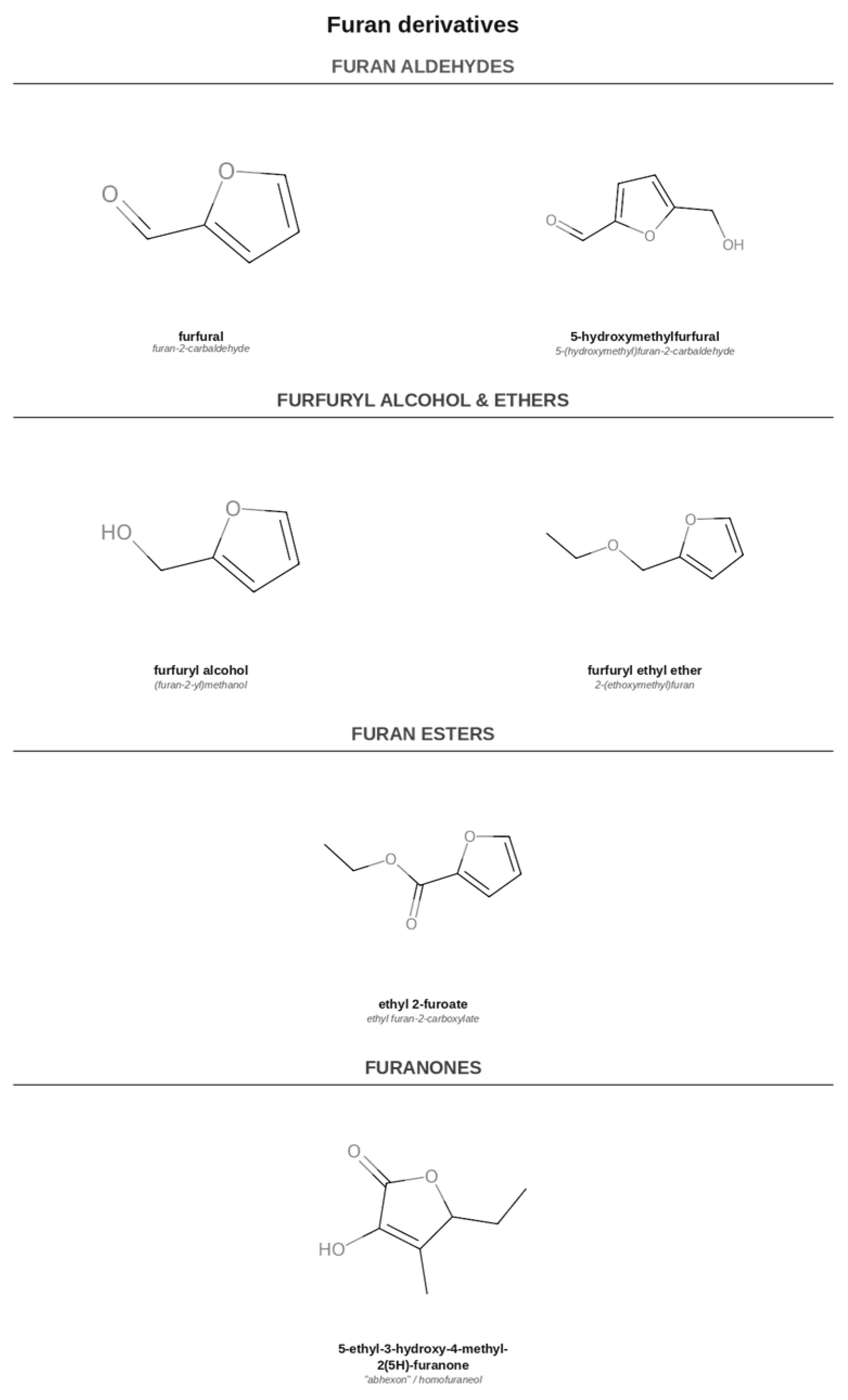
Selected furan derivatives reported in beer production and ageing, including furan aldehydes, furfuryl alcohol and ethers, furan esters, and furanones [33,58,59,60,61,62,63,64,65]. Chemical structures prepared by the authors.

**Figure 4:**
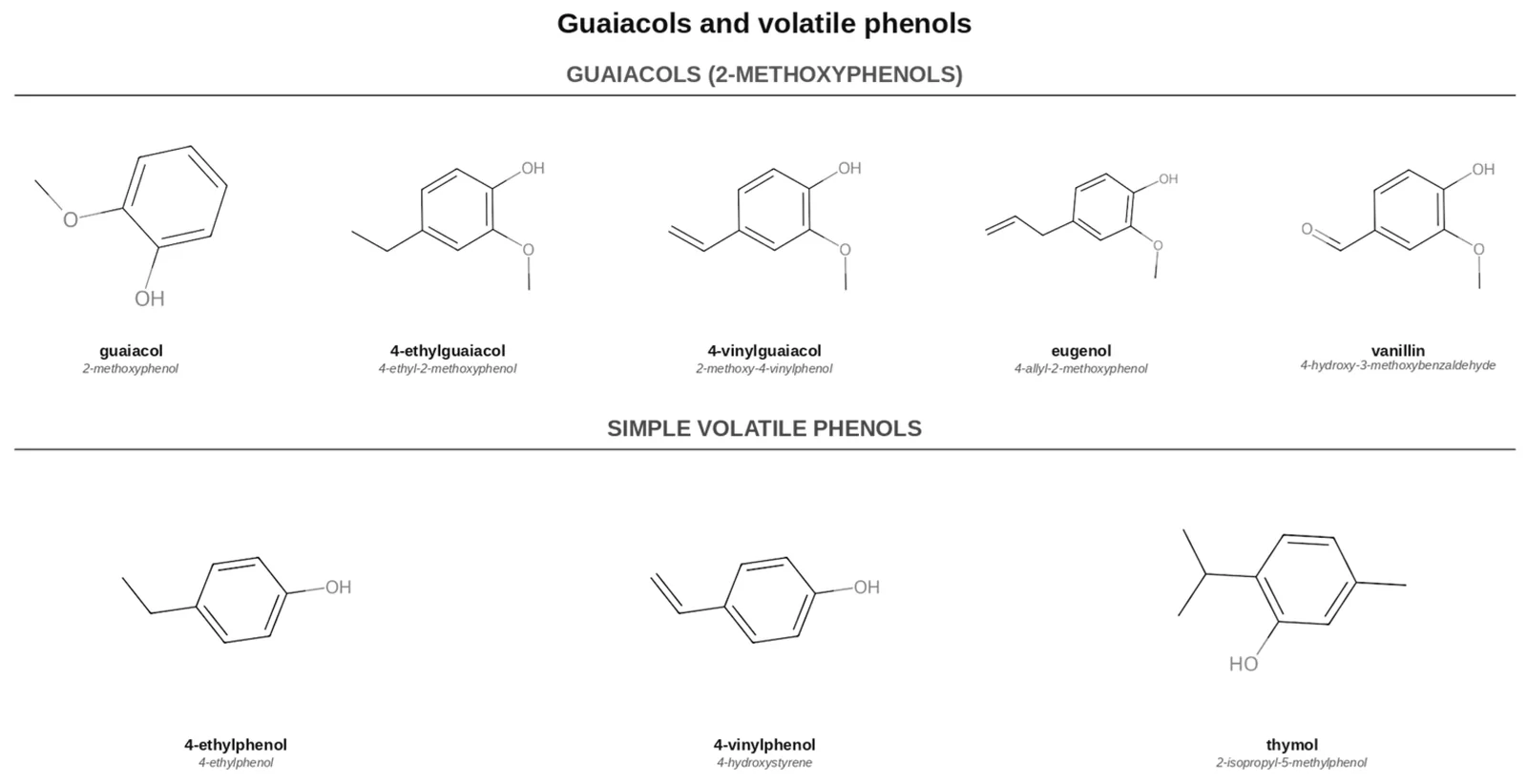
Selected volatile phenols and related aromatic compounds relevant to beer and wood maturation, including guaiacol, substituted guaiacols, eugenol, 4-ethylphenol and 4-vinylphenol [6,9,14,18,33,47,66]. Chemical structures prepared by the authors.

**Figure 5:**
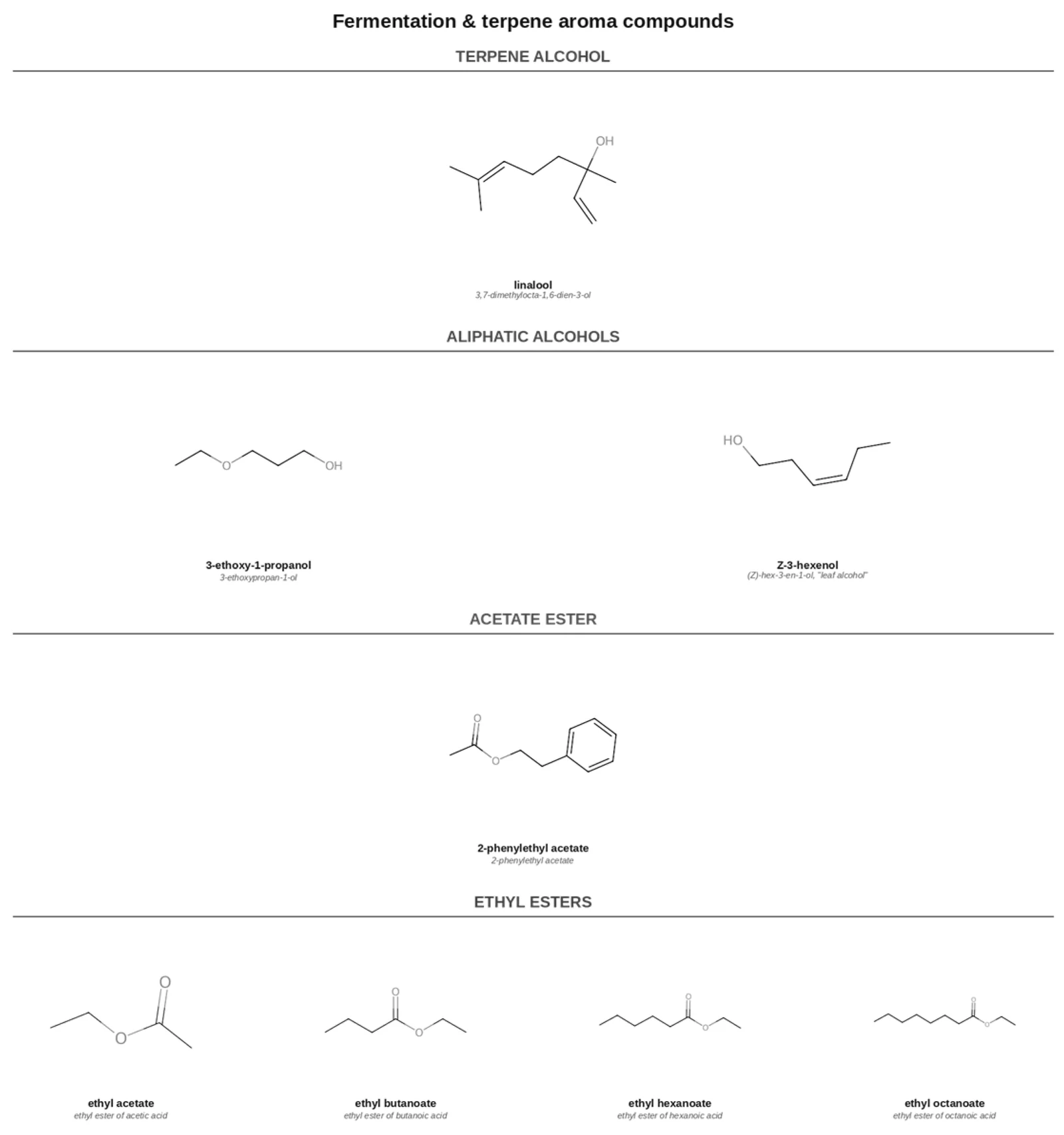
Selected fermentation-, hop- and beer-derived volatile compounds reported to change during wood or barrel maturation, including terpene and aliphatic alcohols and selected acetate and ethyl esters [20,33,36,67,68,69]. Chemical structures prepared by the authors.

**Table 1:** Direct and supplementary evidence on selected wood species relevant to beer maturation.

Wood	Studied System	Main Reported Finding	Evidence/Ref.
Oak (*Quercus* spp.; species as reported in individual studies)	Beer; chips, cubes and barrels	Changes in phenolic compounds, lactones and volatile profile; effects depend on wood origin and process conditions	Direct beer evidence [5,6,7,8,11,14,19,20]
Acacia (*Robinia pseudoacacia*)	Beer; barrels	Lower oak lactones and eugenol than oak; differences in polyphenols, β-glucan and microbial composition	Direct beer evidence [11]
Cherry (*Prunus avium*)	Beer; chips	Short maceration produced only limited changes in volatile composition	Direct beer evidence [5]
Brazil nut (*Bertholletia excelsa*) and other Brazilian woods	Beer; cubes	Changes in maturation congeners and sensory characteristics were reported, but effects were species-specific	Direct beer evidence [16]
Tajuva (*Maclura tinctoria*)	Hydroethanolic model	Higher phenolic extraction than French oak under the investigated model conditions	Model system evidence [17]

**Table 2:** Main technological characteristics and limitations of wood contact systems used in beer maturation.

Wood Contact System	Main Technological Characteristic	Typical Process Implication	Main Limitation of Comparison
Whole barrels	Extraction combined with vessel-related oxygen transfer and potential microbial interactions	Gradual, condition-dependent maturation	Strongly affected by barrel geometry, age, previous use and microbiota
Staves/spirals	Extraction from wood fragments in a non-wooden vessel	Greater control of wood exposure than in barrels	Direct evidence in beer remains limited
Chips/cubes	Predominantly extractive wood–beer contact with relatively high effective surface area	Dose and contact time can be controlled directly	Does not reproduce the full barrel environment
Extracts/essences	Addition of pre-extracted wood-derived compounds	Rapid and reproducible flavor adjustment	Does not reproduce natural wood extraction, oxygen transfer or microbial interactions

**Table 3:** Significance of selected parameters of wood application in beer maturation.

Parameter	Technological Significance	Potential Quality Effect	Main Risk
Wood species	Determines the composition of compounds extracted into beer	Differentiation of the aromatic, phenolic and color profile	Difficult comparability of results between wood species
Degree of toasting	Modifies the degradation products of lignin and hemicellulose	Increased contribution of vanilla, toasted, smoky, spicy or roasted notes	Excessive intensity of roasted, smoky or bitter aromas
Wood chip dose	Determines the amount of material available for extraction	Increased intensity of the woody profile	Dominance of wood over the base beer profile
Contact time	Influences the extent of migration of compounds from wood into beer	Gradual enrichment of the aromatic and flavor profile	Astringency, persistent woody aftertaste, disturbance of sensory balance
Temperature	Influences extraction kinetics and diffusion rate	Acceleration of the extraction of selected compounds	Faster development of undesirable characteristics if the process is not controlled
Alcohol content	Increases the solubility of some phenolic and volatile compounds	Stronger extraction in beers with higher alcohol content	Different responses of low-alcohol and high-alcohol beers
Beer style	Determines the ability to integrate woody notes with the base profile	Better integration in dark, strong, sour and specialty beers	Excessive dominance of wood in pale and delicate beers
Wood chip preparation	Includes control of the quality, origin and cleanliness of the material	Greater repeatability and process safety	Microbiological risk, variability between raw material batches or heterogeneity of toasting degree

**Table 4:** Microbiological evidence relevant to wood use during beer maturation and its applicability to wood chip treatment [11,31,32,35,39].

Evidential Basis	Wood System	Microbial Finding	Conditions	Relevance to Wood Chips
Direct barrel study	Oak and acacia barrels	*P. damnosus* and *B. bruxellensis* dominated; wood-dependent bacterial differences	38 weeks	Indirect for chips
Direct barrel study	New and used oak barrels	Dynamic bacterial and fungal succession	38 weeks	Indirect for chips
Controlled model	Wood-maturation model	Ethanol and iso-α-acids altered community establishment	Controlled model	Mechanistic evidence
Laboratory model	Wooden disk	Reproduced selected barrel-associated microbial trends	Defined 8-species community	Mechanistic evidence
Sanitation evidence	Wooden barrels	Microbial persistence and sanitation challenges	Review evidence	Relevant to risk assessment; not chip-specific

**Table 5:** Major compound groups and analytical measures relevant to wood-associated beer maturation [11,14,19,33,34].

Compound Group/Measure	Examples Reported in Beer	Sensory Relevance	Other Relevance/Limitation
Oak lactones	cis-/trans-oak lactone, (Z)-oak lactone	Coconut, woody, sweet; sensory relevance demonstrated in selected studies	Concentration and sensory effect depend on wood and maturation conditions
Phenolic acids and related phenolics	Gallic, vanillic, syringic, ellagic acids; catechin	Compound-specific; not all are directly aroma-active	Associated with antioxidant activity, bitterness, astringency and colloidal properties
Phenolic aldehydes	Vanillin, syringaldehyde, coniferaldehyde, sinapaldehyde	Vanilla, spicy or woody associations	Sensory contribution depends on concentration and threshold
Furanic compounds	Furfural, 5-HMF, ethyl 2-furoate	Toasted, caramel-like or sweet associations for selected compounds	Detection does not by itself demonstrate sensory causality
Volatile phenols	Eugenol, 4-ethylguaiacol, 4-ethylphenol, 2,6-dimethoxyphenol	Clove, smoky, spicy, phenolic	May originate from wood, malt and/or microbial transformations
Other volatile compounds	Ethyl lactate, ethyl decanoate, ethyl phenylacetate, sesquiterpenes	Compound-dependent	Not a single chemical class; changes may reflect extraction, adsorption or maturation

**Table 6:** Reported lactone concentrations across wood maturation matrices and systems.

Maturation System	Matrix/Style	Target Compound	Reported Concentration	Sensory Impact
Oak barrels (38 weeks)	Strong blond beer	Cis/trans-oak lactone	96.0 µg/L (*cis*), 154.8 µg/L (*trans*)	Coconut, vanilla notes [11]
Port wine oak barrels	Belgian dark strong ale	Trans-oak lactone	Peaked at 50.5 µg/L (week 11)	Woody aromatics [2]
American oak chips (wine)	Model/imperial stout	Whisky lactones	Detected only in oak treatments	Vanilla and coconut nuances [19]

**Table 7:** Evidence used to assess the quality effects of wood chips relative to untreated and barrel-matured beer.

Study/Evidence Type	Design and Control	Wood Treatment/Setup	Main Finding	Key Methodological Limitation
Correia et al. [5]—direct beer-chip study	Ale, lager and porter; untreated controls	Oak/cherry chips; 1 g/L; 30 d	Limited, beer-dependent volatile changes	Short contact and low dose; no barrel comparator
Sterckx et al. [6,7]—beer study + model system	Beer experiment plus beer-like model solutions	Oak chips; extraction variables investigated	Increased monophenols and selected woody/spicy attributes	Model system results cannot be quantitatively equated with finished beer
Cioch-Skoneczny et al. [8]—direct beer-chip study	Porter with untreated controls	American/French oak; 3–7 g/L; fermentation or maturation	Physicochemical and volatile changes; no consistent dose response	Multiple process variables limit simple dose–effect interpretation
Machado et al. [14]—direct beer-chip study	Pale/dark beer	American/French oak; 2 g/L; 21 d	Selected phenolic acids differed; malt effect was stronger overall	Beer matrix confounded interpretation of wood origin effects
Wyler et al. [20]—three-system comparison	Lager; cubes, barrel and untreated control	3 months; 0 °C	Chemical differences; no significant acceptance difference	Container/volume differed; untrained panel; no equivalence test
Bossaert et al. [11]—barrel reference	Conventionally fermented beer	Oak/acacia barrels; 38 weeks	Chemical, sensory and microbial differentiation	Barrel evidence; not direct evidence for chips

**Table 8:** Main beer categories associated with barrel maturation.

Beer Category	Examples Supported by the Reviewed Literature	Main Sensory or Technological Characteristics	Technological Role of Barrel Maturation
Strong, dark and high-alcohol beers	Stout-type beer; high-alcohol beer	Wood, vanilla, toast, spice, smoke, coconut-like notes; integration with intensive beer matrix	Extraction of wood-derived compounds and development of sensory complexity
Sour and mixed-microbiota beers	Sour beer; beers involving microbial succession during wood maturation	Acidity, phenolic notes, ester formation, microbial complexity, risk of volatile acidity or instability	Long-term maturation, microbial interactions and chemical transformation during barrel ageing
Barrel-aged lagers	Barrel-aged lager beer	Modification of volatile and metabolic profile; higher risk of imbalance due to more delicate beer matrix	Controlled development of barrel-derived aroma in a lighter beer matrix
Specialty and craft aged beers	Diverse-aged craft beers; barrel-aged beers assessed by sensory tools	Broad and variable profile depending on base beer, wood contact and maturation conditions	Product differentiation and structured development of sensory complexity

**Table 9:** Operational and cost components relevant to comparative assessment of wood chips and barrels.

Cost or Operational Component	Wood Chips or Wood Fragments	Barrels	Current Evidence and Limitations
Purchase of wood material	Generally lower unit cost; smaller wood fractions can be used	High purchase cost, particularly for new or specialized barrels	Mainly qualitative evidence; prices vary by wood, origin and supplier [104]
Process vessel	Can be applied in existing maturation tanks	Requires dedicated wooden vessels	Operational advantage is plausible, but brewery-scale comparisons are limited [21,102]
Process duration	Commonly days to weeks, although longer treatments are possible	Commonly months and, in some products, years	Direct comparison is limited by differences in beer and process conditions [5,8,11,13,14,19,20,32,33,67]
Storage requirements	Tank volume is occupied for the duration of treatment, but no separate barrel storage area is required	Requires barrel storage, access space and handling infrastructure	Rarely quantified in terms of area per unit of annual production
Product losses	Beer may be retained by chips or lost during removal and separation	Evaporation, wood absorption, leakage and residual beer may occur	Very limited quantitative data for beer [103,104]
Labour requirements	Material receiving, preparation, dosing, sampling and removal.	Filling, topping, sampling, transfer, cleaning, inspection and handling.	Different task structures; comparative labour-time data are unavailable.
Sanitation	Requires control of chip microbiology and tank hygiene	Porous barrel structure complicates cleaning and disinfection	Better documented for barrels than for fragmented wood [39,71].
Repeatability	Repeatability may be improved through standardization of raw material and process parameters.	Affected by barrel history and inter-barrel variability.	Comparative repeatability requires replicated pilot- or brewery-scale validation.
Reuse	Possible, but extractive capacity declines with successive cycles	Multiple uses are possible; barrels may be regenerated or modified	Evidence mainly from wine and spirits [34,106,110,111]
End-of-life management	Potential biomass feedstock or secondary material	Regeneration, resale, dismantling or material recovery	Environmental benefits require life-cycle assessment [104,113,114,115,116,117,118,119]

The table does not establish that one technology is universally less expensive. It identifies the variables that should be included in a brewery-specific techno-economic assessment.

## Data Availability

The original contributions presented in this study are included in the article. Further inquiries can be directed to the corresponding authors.
